# Flavor Formation in Goat Meat: A Lipid-Centric Comparative Study of High-Altitude and Low-Altitude Breeds

**DOI:** 10.3390/foods15050855

**Published:** 2026-03-04

**Authors:** Jingjing Li, Yidan Xu, Zhenzhen Zhang, Yanqiu Huang, Nan Zhang, Wangjie Zhaxi, Zhaxi Danba, Duoji Jinmei, Tianzeng Song, Wangsheng Zhao

**Affiliations:** 1 College of Life Science and Agri-Forestry, Southwest University of Science and Technology, Mianyang 621010, China; jingjingyi11@126.com (J.L.); zhangzhen@swust.edu.cn (Z.Z.); 17738595262@163.com (Y.H.); 2 Xizang Animal Husbandry Station, Lhasa 850000, China; nanan_cc008@126.com; 3 The Service Station of Agricultural and Animal Husbandry Technical of Bange County, Nagqu 852500, China; 18208007776@163.com; 4 Nagqu Technology Research and Promotion Center for the Agriculture and Animal Husbandry (Grassland) of Nagqu, Nagqu 854000, China; 18239988000@163.com; 5 The Service Station of Agricultural and Animal Husbandry Technical of Anduo County, Nagqu 853400, China; duojijinmei1234@163.com; 6 Institute of Animal Science, Xizang Academy of Agricultural and Animal Husbandry Science, Lhasa 850009, China

**Keywords:** goat, volatile organic compounds, glycerophospholipids, gas chromatography–mass spectrometry

## Abstract

Flavor is a pivotal determinant of goat meat quality, influenced by multiple factors. This study investigated flavor formation from a lipid perspective by comparing two distinct breeds at two years old and fed the same diet: the high-altitude Xizang goat (XG; *n* = 6, 26.23 ± 0.72 kg), renowned for its unique meat flavor, and the low-altitude meat-type Jianzhou big-ear goat (JBG; *n* = 6, 63.93 ± 0.98 kg). Lipid profiles were analyzed using liquid chromatography–tandem mass spectrometry (LC–MS/MS), and flavor variations were assessed using gas chromatography–mass spectrometry (GC–MS). We identified 630 significantly differential lipids (VIP > 1, *p* < 0.05) between the breeds. The XG group exhibited a distinct lipid composition characterized by a higher proportion of glycerophospholipids (45.1%) and the upregulation of specific species such as PC (13:0_16:0) and PE(16:0_20:5), whereas glycerolipids were markedly more abundant in JBG (24.3%) than in XG (6.4%). A total of 14 key volatile organic compounds (VOCs) were identified as potential drivers of flavor divergence based on the criteria of |log_2_(fold change)| ≥ 1, VIP > 1, *p* < 0.05 and rOAV ≥ 1. Correlation networks revealed significant positive associations (r > 0.8, *p* < 0.05) between several upregulated glycerophospholipids—including PC (13:0_16:0), PE(16:0_20:5), PE(20:5_16:1), PMeOH(16:0_22:4), and PS(18:2_20:5)—and fruity esters such as ethyl heptanoate and butyl butyrate in XG meat, directly contributing to its more intense fruity sensory profile. Collectively, this study demonstrated that the phospholipid-rich lipidome of high-altitude XG served as a key substrate for generating fruity esters, which fundamentally distinguishes its more complex and preferred sensory profile from the triglyceride-dominated lipidome of JBG meat. These findings establish a potential molecular link between lipid composition and meat flavor, providing a biochemical explanation for traditional flavor preferences and highlighting the importance of lipid metabolism in determining the quality of goat meat.

## 1. Introduction

Advances in living standards and growing health consciousness have significantly increased consumer demand for meat with a more desirable and complex sensory profile, including enhanced flavor intensity, juiciness, and overall palatability [1,2,3]. Flavor, a key determinant of meat acceptability, is closely influenced by lipid metabolism [4]. In particular, intermuscular fat (IMF) plays a critical role in determining the flavor profile of goat meat [5]. Its primary constituents—triacylglycerols and phospholipids—are fundamental to flavor formation [4,6,7]. During thermal processing, the phospholipids, which are rich in polyunsaturated fatty acids (PUFAs), undergo oxidation to generate key volatile compounds that define the characteristic meat flavor [8,9]. Moreover, the level of IMF directly influences the release and perception of these flavor volatiles: a higher IMF content promotes their formation and dispersion, while a lower content limits their release [6,10]. Thus, IMF content is a key factor in achieving the desired sensory quality of cooked goat meat.

Given that IMF is the key component contributing to flavor, its content and composition are significantly regulated by environmental factors [5]. Among these, altitude plays a defining role. We focus on altitude as a primary environmental regulator because it integrates critical stressors—such as hypoxia and temperature—which systemically altered metabolism and nutrient deposition [11,12]. These altitude-driven adaptations lead to distinct differences in nutritional composition, amino acid profiles, and fatty acid (FA) patterns in mutton [11,13]. For instance, meat from high-altitude regions exhibited a higher protein content and a more abundant amino acid profile [14], while altitude-driven adaptations in lipid metabolism may further affect the flavor profiles of goat meat [15]. The Jianzhou big-ear goat (JBG) is a typical low-altitude breed valued for its high growth rate, disease resistance, and superior meat production. In contrast, the Xizang goat (XG), indigenous to the Tibetan Plateau at elevations around 4000 m, is reported to produce meat with a more intense umami taste and a distinct flavor profile. These sensory attributes are supported by studies reporting substantial levels of key flavor precursors in XG meat, such as free amino acids (e.g., glutamic acid) and a significant proportion of PUFAs (e.g., linoleic and linolenic acid) [16]. Combined with lower levels of harmful metal elements (0.20 ± 0.02 (Pb), 0.57 ± 0.06 (Cr), 4.31 ± 0.18 (Cd), 5.02 ± 0.54 (Ni), and 0.02 ± 0.01 (Co) mg/kg) [17], these traits contribute to its recognition for quality [18,19]. However, the specific lipid and flavor-related metabolites underlying the distinctive flavor of XG meat remain poorly understood, particularly in direct comparison with high-performance meat-type breeds such as JBG.

To address this knowledge gap and elucidate the potential molecular link between lipid composition and meat flavor, lipidomics and metabolomics offer powerful tools. Lipidomics provides a systematic approach to comprehensively profile lipid compositions and their variations in biological systems [20,21] and has been applied to explore lipid molecules associated with meat quality and flavor in goats [22]. Similarly, flavor metabolomics, especially through techniques such as gas chromatography–mass spectrometry (GC–MS), enables detailed characterization of volatile flavor compounds, offering valuable insight into the sensory attributes of meat [23]. This study employed an integrated lipidomics and flavor metabolomics approach to compare locally adapted goat breeds from contrasting altitudes, with the aim of identifying the key lipids and volatile metabolites that contribute to their distinctive meat flavors.

Therefore, this study specifically aimed to compare the lipidomic and metabolomic profiles of the *longissimus dorsi* (LD) muscle between the high-altitude XG and the low-altitude JBG. By integrating lipidomic and flavor metabolomic data, we sought to pinpoint the crucial lipid species and volatile metabolites responsible for the unique sensory attributes of each breed’s meat. The findings are expected to provide a scientific basis for evaluating goat meat quality and to offer deeper insights into the sensory characteristics of goat breeds from different geographical origins.

## 2. Materials and Methods

### 2.1. Ethics Statement

All experimental procedures in this study were conducted in accordance with the Guidelines for Experimental Animals established by the Ministry of Science and Technology of the People’s Republic of China. The protocol was approved by the Animal Ethics Committee of Southwest University of Science and Technology (Permit ID: L2022026, approval Date: 15 March 2022).

### 2.2. Experimental Animals and Sample Preparation

The Xizang goats (XG) and Jianzhou big-ear goats (JBG) were reared in their respective native regions. XG were sourced from a local farm in Lhasa, Xizang, China (latitude: 40°34′20.61″ N, longitude: 110°31′22.48″ E; altitude: 3650 m). JBG were sourced from a local farm in Jianyang, Sichuan, China (latitude: 30°23′43.85″ N, longitude: 104°32′41.74″ E; altitude: 500 m). This design allows for a comparison of breeds under their typical rearing environments. A total of twelve healthy, intact male goats (six per breed) were used. To minimize the influence of close genetic relatedness, all selected individuals were confirmed not to be full- or half-siblings. The sample size for the omics analyses was determined based on common practices in similar omics studies investigating meat quality [24,25,26]. Within each breed, goats had similar body weights. All goats were weaned at 60 days of age under standard farm conditions and raised until approximately two years old, which is considered the standard market-ready “finished” age for these breeds [27]. All goats were housed individually in indoor pens (1.5 × 1.0 m) at their respective farms and fed the same finishing diet twice daily (08:00 and 17:00), formulated according to the “Meat Sheep Feeding Standards” (NY/T 816–2004) [28]; the composition and nutritional level are shown in Table S1.

Prior to slaughter, goats were fasted for 16 h and then weighed (XG: 26.23 ± 0.72 kg; JBG: 63.93 ± 0.98 kg). The large difference in mature weight is a known breed characteristic [27,29]. In November 2024, all goats were transported a short distance (<10 km) to their respectively local abattoir and processed following identical protocols: electrical stunning, exsanguination, and the slaughterhouse facilities complied with the guidelines of the China Council on Animal Care. Immediately after slaughter, muscle samples were collected from the *longissimus dorsi* (LD) at the level of the 5th lumbar vertebra. Visible external fat and connective tissue were trimmed. A sample of approximately 10 g was excised from the core of the LD muscle (approximate temperature at excision: 37–39 °C). Samples were immediately snap-frozen in liquid nitrogen and stored at –80 °C until analysis. Climatic data, representing the characteristic annual conditions of the respective regions, were obtained from https://www.weather-atlas.com/zh (accessed on 13 January 2025).

### 2.3. Extraction of Lipid and Liquid Chromatography–Tandem Mass Spectrometry (LC–MS/MS) Analysis

Lipid extraction was performed according to a modified MTBE method [16]. Briefly, approximately 20 mg of powdered tissue (ground under liquid nitrogen) was weighed. Then, 1 mL of the lipid extraction solvent (methyl tert-butyl ether/methanol, 3:1, *v*/*v*), which was pre-supplemented with an internal standard mixture, was added. The internal standards included representative lipid species from major classes, such as Cer(d18:1/4:0), PC(13:0/13:0), PE(12:0/12:0), and TG(17:0/17:0/17:0). The mixture was vortexed vigorously for 15 min. After the addition of 200 μL of ultrapure water, the sample was vortexed for 1 min and centrifuged at 12,000× *g* for 10 min at 4 °C. A 200 μL aliquot of the upper organic layer was collected and dried under a gentle nitrogen stream. The dried extract was reconstituted in 200 μL of acetonitrile/isopropanol (1:1, *v*/*v*) for LC-MS/MS analysis.

LC analyses were performed on an ExionLC AD UPLC system (SCIEX, Framingham, MA, USA). Lipid separation was performed on a Thermo Accucore™ C30 column (2.1 mm × 100 mm, 2.6 µm, Thermo Fisher, Waltham, MA, USA) maintained at 45 °C with a 2 µL injection volume, using a binary mobile phase system consisting of (A) acetonitrile/water (60:40, *v*/*v*) and (B) acetonitrile/isopropanol (10:90, *v*/*v*), both supplemented with 0.1% formic acid and 10 mmol/L ammonium formate, purchased from Sigma-Aldrich. A gradient elution was performed at a flow rate of 0.35 mL/min as follows, 0 min for A/B (80:20, *v*/*v*), 2 min for (70:30, *v*/*v*), 4 min for (40:60, *v*/*v*), 9 min for (15:85, *v*/*v*), 14 min for (10:90, *v*/*v*), 15.5 min for (5:95, *v*/*v*), 17.3 min for (5:95, *v*/*v*), 17.3 min for (80:20, *v*/*v*), and 20 min for (80:20, *v*/*v*). Mass spectrometry detection was conducted using electrospray ionization (ESI) in both positive and negative modes.

Mass spectrometry detection was conducted using electrospray ionization (ESI) in both positive and negative modes on a SCIEX QTRAP^®^ 8600+ triple quadrupole mass spectrometer. Lipid identification was based on a pre-established Metware database, matching the retention time and precursor–product ion pairs of each analyte against database entries. Quantification was subsequently carried out using the multiple reaction monitoring (MRM) mode. The source temperature was 500 °C; ion spray voltages were set at 5500 V (positive) and –4500 V (negative). Gas pressures for ion source gas 1 (GS1), ion source gas 2 (GS2), and curtain gas (CUR) were 45, 55, and 35 psi, respectively. Collision-activated dissociation (CAD) was set to medium.

For data processing and analysis, lipid identification, peak extraction, peak alignment, and quantification analysis were evaluated using LipidSearch software (v4.1; Thermo Fisher Scientific, Waltham, MA, USA). The identified compounds were classified using the Lipid Maps database. The identified lipids were classified according to the Lipid Maps database. Multivariate statistical analyses, including principal component analysis (PCA) and orthogonal partial least squares-discriminant analysis (OPLS-DA), were performed using the ropls package (v1.6.2) in R. The robustness of the OPLS-DA model was validated by 200 permutation tests. Differential lipids were screened with the criteria of variable importance in projection (VIP) > 1 from the OPLS-DA model and a Student’s *t*-test *p* value < 0.05. Kyoto Encyclopedia of Genes and Genomes (KEGG) pathway enrichment analysis was performed on the differential lipids (http://www.kegg.jp/kegg/pathway.html, accessed on 25 April 2025).

### 2.4. Analysis of Volatile Compounds

Volatile compounds were analyzed by headspace solid-phase microextraction coupled with gas chromatography–mass spectrometry (HS-SPME/GC–MS) as previously described with modifications [30]. Briefly, approximately 0.08 g of powdered muscle tissue was weighed into a 20 mL headspace vial. Then, 0.2 g of NaCl and 20 μL of an internal standard solution (3-Hexanone-2,2,4,4-d4) were added. The HS-SPME extraction was performed using an automated system (CTC Analytics AG, Zwingen, Switzerland). The sample was incubated at 100 °C for 5 min with agitation. Volatile compounds were then adsorbed onto a 120 μm DVB/CWR/PDMS fiber for 15 min at the same temperature. The fiber was subsequently desorbed in the GC inlet at 250 °C for 5 min.

GC–MS analysis was conducted on an Agilent system equipped with a DB-5MS capillary column (30 m × 0.25 mm × 0.25 μm). High-purity helium was used as the carrier gas at a constant flow rate of 1.2 mL/min. The inlet temperature was 250 °C. The programmed warming routine was as follows: 40 °C for 3.5 min, 10 °C/min to 100 °C, 7 °C/min to 180 °C, and finally 25 °C/min to 280 °C, holding for 5 min. The mass spectrometer operated in electron impact (EI) mode at 70 eV. The ion source and quadrupole temperatures were 230 °C and 150 °C, respectively. For compound identification, preliminary full-scan (SCAN) mode was performed on pooled quality control (QC) samples. Chromatographic peaks were matched against the NIST 2020 spectral library using a minimum similarity threshold of 80%. In combination with retention index calibration [31], a self-built database was constructed, containing the confirmed retention time and 2–3 diagnostic fragment ions for each target compound [32]. In formal sample analysis, all acquisitions were conducted exclusively in selected ion monitoring (SIM) mode. Each target ion was monitored within a dedicated time window aligned with its expected elution order. Select quantitative ions for integration and calibration using MassHunter software (version B.08.00) to enhance quantitative accuracy.

Semiquantitative analysis was performed using 3-Hexanone2,2,4,4-d4 as an internal standard, and the following formula was used:

(1) $$X_{i}\;=\;\frac{V_{s}\;\times \;C_{s}}{M}\;\times \;\frac{I_{i}}{I_{s}}\;\times \;10^{-3}$$

where X_i_ (μg·g^−1^) is the concentration of compound i, vs. (μL) represents the volume of interstandard, C_s_ (μg·ml^−1^) represents the concentration of the interstandard, M (g) represents the weight of the lamb sample, I_s_ represents the peak area of the interstandard, and I_i_ is the peak area of any flavor compound in the sample.

The levels of volatile compounds obtained by HS-SPME-GC-MS were expressed as the relative content. Differential volatile compounds were determined using the criteria of |log_2_(fold change)| ≥ 1, VIP > 1, and *p* < 0.05. The relative odor activity value (rOAV) method was calculated to evaluate the contribution of each compound to the overall flavor [20], which was correlated with the concentration of the compound, and odor thresholds in the water (μg·g^−1^) were obtained from odor databases and literature [33]. A volatile compound is considered a key contributor to the overall flavor profile when rOAV > 1, indicating that its concentration in the sample meets or exceeds its sensory detection threshold [34]. The rOAV was calculated as follows:

(2) $${\mathrm{rOAV}}_{i}\;=\;\frac{C_{i}}{T_{i}}$$

where rOAV_i_ denotes the relative odor activity value of compound i, C_i_ (μg·g^−1^) is the relative content of compounds, and T_i_ represents the odor threshold value of compound.

A correlation network between sensory flavor characteristics and volatile metabolites was visualized using Cytoscape (v3.10.3). KEGG pathway enrichment analysis was performed on differential volatile metabolites.

### 2.5. Statistical Analysis and Correlation Analysis

Data were expressed as mean ± standard error of the mean. Statistical analyses between the two breed groups were assessed using Student’s *t*-test in SPSS software (version 26.0) (SPSS, Inc., Chicago, IL, USA). A *p* value < 0.05 was considered a significant difference. Post hoc statistical power (1 − β) was calculated using the G*Power (version3.1.9.7), based on the observed effect sizes, sample size (*n* = 6, per group), and α = 0.05. Correlation analyses between lipid species and volatile flavor compounds were performed using Spearman’s rank correlation, and heatmaps were generated in R. Mantel tests were conducted to assess the correlation between lipid profiles and flavor attributes using the Metware Cloud platform (https://cloud.metware.cn, accessed on 25 April 2025).

## 3. Results

### 3.1. Climate Change in the Native Habitat Environment of Xizang and Jianyang

**Figure 1:**
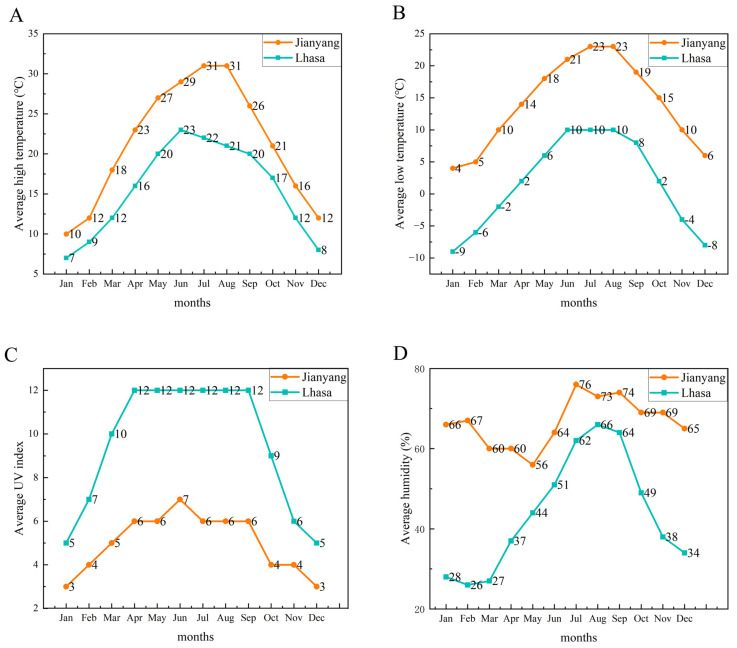
Comparison of representative annual climatic profiles in the native habitat environment of Xizang goats and Jianzhou big-ear goats. (**A**) Comparison of changes in average high temperatures. (**B**) Comparison of changes in average low temperatures. (**C**) Comparison of changes in average UV index. (**D**) Comparison of changes in average humidity.

Climatic data from a complete annual cycle were collected for the native habitat of each goat breed to characterize the fundamental features of their long-term adaptive environment (Figure 1A–D). Although the raising period of the experimental goats spanned approximately 22 months, the annual climatic pattern represents the primary environmental factor shaping their long-term adaptive phenotypes, including muscle metabolites. The data revealed that the Lhasa area (habitat of Xizang goats) exhibited an approximately 9 °C lower average temperature and 23% lower average humidity compared to the Jianyang area (habitat of Jianzhou big-ear goats). The average ultraviolet (UV) index in Lhasa was substantially higher, approximately 1.9-fold greater than that in Jianyang.

### 3.2. Lipid Composition in Two Goat Breeds from Different Altitudes

**Figure 2:**
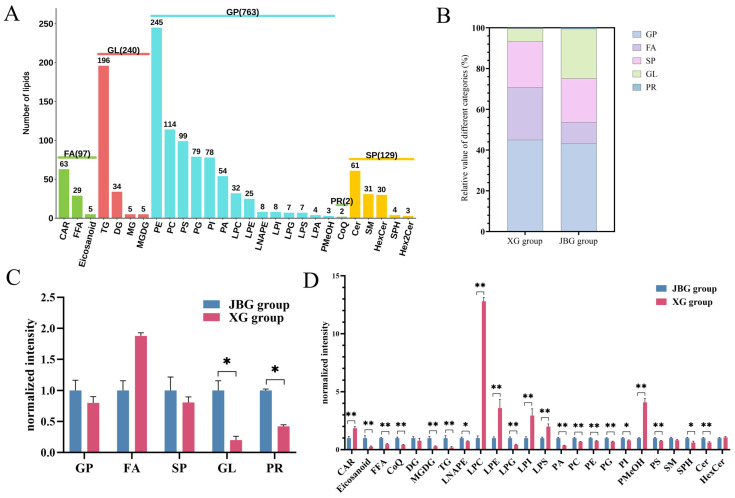
Comparison of lipidomic profiles in longissimus dorsi muscles from Xizang goats and Jianzhou big-ear goats. (**A**) Classification statistics of lipid composition. FA: fatty acid; GL: glycerolipid; GP: glycerophospholipid; PR: prenol lipid; SP: sphingolipid; CAR: cholesteryl ester; FFA: free fatty acid; Eicosanoid: eicosanoid; TG: triacylglycerol; DG: diacylglycerol; MG: glyceryl monostearate; MGDG: monogalactosyldiacylglycerol; PE: phosphatidylethanolamine; PC: phosphatidylcholine; PS: phosphatidylserine; PG: phosphatidylglycerol; PI: phosphatidylinositol; PA: phosphatidic acid; LPC: lyso-phosphatidylcholine; LPE: lyso-phosphatidylethanolamine; LNAPE: lyso-N-acyl phosphatidylethanolamine; LPI: lyso-phosphatidylinositol; LPG: lyso-phosphatidylglycerol; LPS: lyso-phosphatidylserine; LPA: lysophosphatidic acid; PMeOH: phosphatidylmethanol; CoQ: coenzyme Q; Cer: ceramide; SM: sphingomyelin; HexCer: Hexosylceramide; SPH: sphingosine; Hex2Cer: Lactosylceramide. (**B**) The relative values of different categories of lipids. (**C**) Comparisons of lipid relative intensities of the first classification between XG and JBG groups. (**D**) Comparisons of lipid relative intensities of subclasses between XG and JBG groups. * *p* < 0.05, ** *p* < 0.01.

LC-MS/MS analysis was conducted to compare the lipid profiles of LD muscle between the high-altitude XG and the low-altitude JBG. The liquid chromatogram of the quality control sample is presented in Figure S1. A total of 1229 lipid molecules were identified and categorized into 5 major classes and 27 subclasses (Figure 2A). Triglyceride (TG), phosphatidylethanolamine (PE) and phosphatidylcholine (PC) were identified as the three most abundant lipid subclasses (Figure 2A).

The relative composition of lipid classes in the LD muscle of XG and JBG is shown in Figure 2B. In the XG group, glycerophospholipids (GP, 45.1%) constituted the largest proportion, followed by fatty acids (FA, 25.7%), sphingolipids (SP, 22.5%), glycerolipids (GL, 6.4%), and prenol lipids (PR, 0.3%). In contrast, the JBG group exhibited a different distribution: GP (43.2%), GL (24.3%), SP (21.5%), FA (10.5%), and PR (0.5%). Comparative analysis revealed significant differences in the relative abundance of several lipid classes and subclasses between XG and JBG groups (*p* < 0.05). At the major class level, the JBG group exhibited significantly higher levels of GL and PR, with GL abundance being approximately 4.96-fold higher in JBG compared to XG group (*p* < 0.05) (Figure 2C). The post hoc power of GL and PR was 0.99 and 1, respectively. These high power values (>0.80) indicate that the sample size used in this study provided a very low probability of a Type II error for these comparisons, lending strong statistical confidence to the authenticity and repeatability of these specific findings. At the subclass level, there was no significant difference in DQ, SM, and Hexcer between two groups (*p* > 0.05). The post hoc power of DQ, SM, and Hexcer was 0.15, 0.47, and 0.09. The non-significant result (*p* > 0.05) and low power (power < 0.2) prevent definitive conclusions regarding the presence or absence of an effect. The XG group demonstrated significantly higher relative intensities of carnitine (CAR), lysophosphatidylcholine (LPC), lysophosphatidylethanolamine (LPE), lysophosphatidylinositol (LPI), lysophosphatidylserine (LPS), and phosphatidylmethanol (PMeOH). Conversely, the JBG group contained significantly higher levels of oxidized lipids (Eicosanoid), free fatty acid (FFA), ceramide (Cer), sphingosine (SPH), coenzyme Q (CoQ), monogalactosyldiacylglycerol (MGDG), TG, N-acyl-lysophosphatidylethanolamine (LNAPE), lysophosphatidylglycerol (LPG), phosphatidic acid (PA), PC, phosphatidylethanolamine (PE), phosphatidylglycerol (PG), phosphatidylinositol (PI), and phosphatidylserine (PS) (*p* < 0.05) (Figure 2D). For instance, TG levels were increased by approximately 4.28-fold in JBG relative to XG group. The post hoc power of these parameters was all >0.8, suggesting that the results were authenticity and repeatability. These results indicate distinct lipidomic profiles between the two goat breeds from different altitudes, which may contribute to differences in flavor characteristics of their meat.

**Figure 3:**
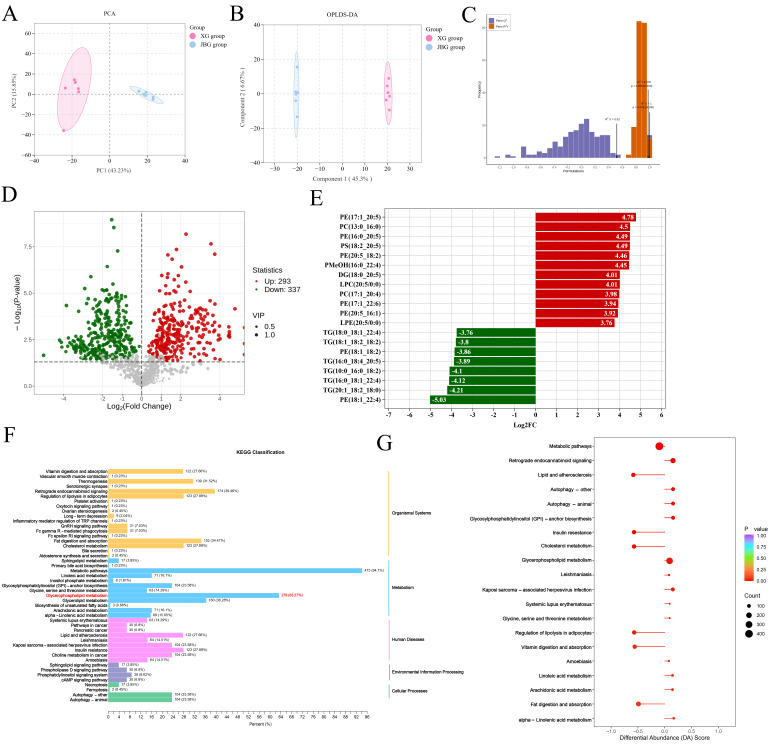
Differential lipid screening and analysis. (**A**) PCA score plots of total lipids between XG and JBG groups. (**B**) OPLS-DA score plots of total lipids between XG and JBG groups. (**C**) OPLS-DA model validation. (**D**) Volcano plots for differential lipids between XG and JBG groups. Each point in the volcano map represents a lipid, with green points representing downregulated differential lipids, red points representing upregulated differential lipids, and gray points representing lipids that are detected but not significantly different. (**E**) Bar chart of the top 20 differential lipids. (**F**) KEGG annotation results of differential lipids. Red indicates that 279 differential lipids were significantly enriched in the glycerophospholipid metabolism pathway. (**G**) KEGG functional difference abundance score map.

To further investigate the lipid differences between breeds from different altitudes, we performed PCA and OPLS-DA. Both score plots revealed distinct separation between the XG and JBG groups (Figure 3A,B). A permutation test (200 repetitions) confirmed the model’s robustness and reliability, yielding R^2^Y = 1, Q^2^ = 0.978, and R^2^X = 0.52 (Figure 3C). A total of 630 significantly differential lipids (293 upregulated, 337 downregulated) were screened based on the thresholds of VIP > 1 and *p* < 0.05 (Figure 3D). Among the top 20 differential lipids, the upregulated lipids in XG group were primarily composed of glycerophospholipids, including LPC(20:5/0:0), LPE(20:5/0:0), PC(13:0_16:0), PC(17:1_20:4), PE(16:0_20:5), PE(20:5_16:1), PE(20:5_18:2), PE(17:1_20:5), PE(17:1_22:6), PMeOH(16:0_22:4), and PS(18:2_20:5). The downregulated lipids were predominantly triglycerides, such as TG(10:0_16:0_18:2), TG(16:0_18:1_22:4), TG(16:0_18:4_20:5), TG(18:0_18:1_22:4), TG(18:1_18:2_18:2), and TG(20:1_18:2_18:0) (Figure 3E).

KEGG pathway enrichment analysis was performed to investigate lipid metabolic pathway variations between the two breeds reared at different altitudes. A total of 279 differential lipids were significantly enriched in the glycerophospholipid metabolism pathway (Figure 3F). Furthermore, the activity of the glycerophospholipid metabolism pathway was notably enhanced in the XG group compared to the JBG group (Figure 3G). These findings suggested that the distinct glycerophospholipid composition, resulting from the combined influence of genetic background and environmental adaptation, was possibly the potential factor contributing to the differences in meat flavor between the two breeds.

### 3.3. The Volatile Organic Compound (VOC) Profiles

#### 3.3.1. Comparative Analysis of VOCs Between XG and JBG Groups

To comprehensively assess the metabolic profiles of the XG and JBG groups from the two breeds and discern the variations in aroma between goats, metabolite analysis was conducted using HS-SPME-GC-MS. Figure S2A presents the total ion chromatogram (TIC) of pooled quality control (QC) samples, with the solvent front (0–3 min) removed for clarity. The prominent peak typically observed at 2–3 min in raw TICs originated from residual n-hexane used to prepare the internal standard solution (3-hexanone-2,2,4,4-d_4_) and does not represent endogenous meat volatiles. Figure S2B shows a representative mass spectrum of butyl butyrate, illustrating how compound identification was supported by spectral matching against reference libraries. It should be emphasized that quantification was not based on the TIC but on extracted ion chromatograms (XICs) of characteristic fragment ions for each target compound and the internal standard. In the XIC, the internal standard appeared as a sharp, symmetric, and baseline-resolved peak (Figure S3). The relative standard deviation (RSD) of the internal standard response in quality control (QC) samples was approximately 8%, confirming high instrumental stability and supporting the reliability of our semi-quantitative comparisons between groups.

**Figure 4:**
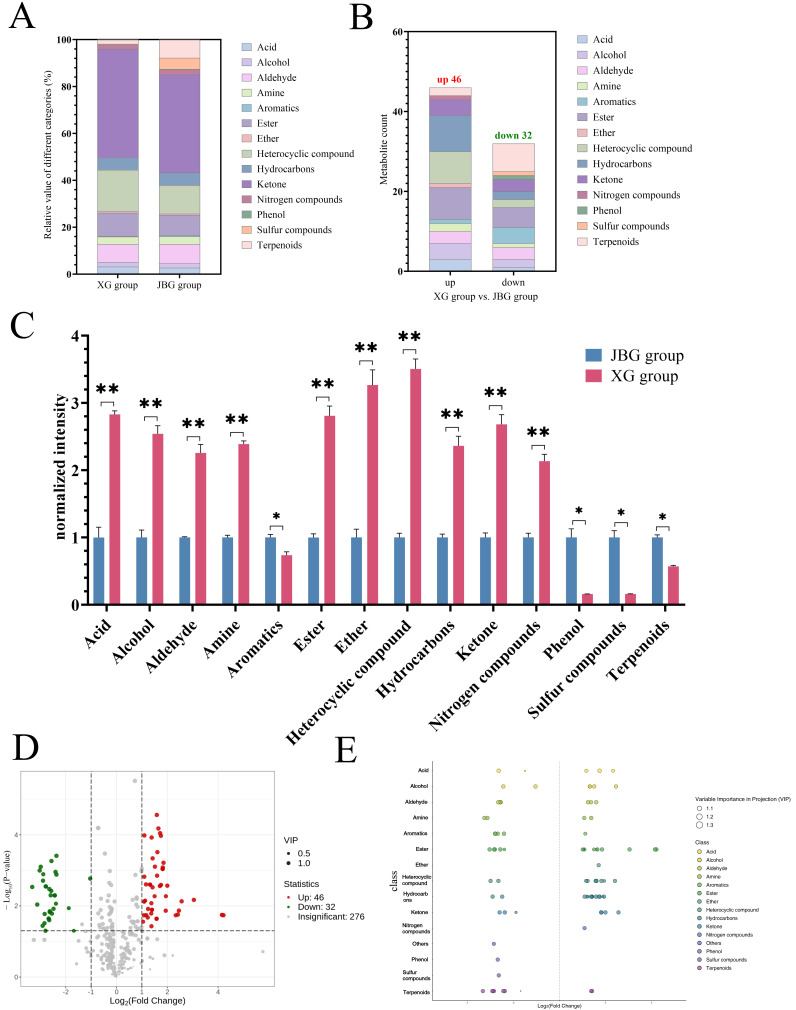
Comparative analysis of volatile organic compounds (VOCs) between XG and JBG groups. (**A**) The relative value of different categories of VOCs. (**B**) Categories and counts of differential VOCs. (**C**) The relative intensities of different classes of VOCs between XG and JBG groups. (**D**) Volcano plots for differential VOCs between XG and JBG groups. Each point in the volcano map represents a VOC, with green points representing downregulated differential VOCs, red points representing upregulated differential VOCs, and gray points representing VOCs that were detected but not significantly different. (**E**) Scatter plot of differential VOCs between HG and LG groups. The horizontal axis represents the logarithm of the relative content difference multiple (log_2_FC) of a certain substance in two groups. The larger the absolute value of the horizontal axis, the greater the content difference in the substance. * *p* < 0.05, ** *p* < 0.01.

A total of 354 VOCs were identified in the current experiment. Detailed information of these VOCs is summarized in Table S2. The composition of VOCs in the LD muscles of the XG and JBG groups is shown in Figure 4A. Ketones constituted the predominant class of volatile flavor compounds in both breeds (Figure 4A). A total of 78 differential VOCs were selected based on above screening criteria, with hydrocarbons having the highest number of differential VOCs and terpenoids having the highest number of downregulated VOCs (Figure 4B). The XG group exhibited significantly higher levels of acids, alcohols, aldehydes, esters, and ketones, among others. Specifically, aldehydes were increased approximately 1.26-fold in XG relative to JBG. Conversely, the JBG group showed significantly higher contents of terpenoids, aromatics, sulfur compound, and phenols, while terpenoid levels were approximately increased by 75% (Figure 4C). Volcano plots revealed the upregulation of 46 differential VOCs and the downregulation of 32 differential VOCs (Figure 4D). Comparative analysis revealed significant differences in the relative contents of multiple volatile categories between the two groups (*p* < 0.05, Figure 4C). The XG group exhibited significantly higher levels of acids, alcohols, aldehydes, amines, esters, ketones, ethers, hydrocarbons, heterocyclic compounds, and nitrogen compounds. In contrast, the JBG group showed significantly higher contents of terpenoids, aromatics, phenols, and sulfur compounds (*p* < 0.05) (Figure 4C). Figure 4E further illustrates the differences in the relative abundance of differential volatile flavor metabolite classes between the two sample groups. These results demonstrated a distinct volatile metabolite profile in the meat of the two goat breeds, which likely contributes to their differing sensory and flavor characteristics.

#### 3.3.2. Identification of Key Flavor-Active VOCs

**Figure 5:**
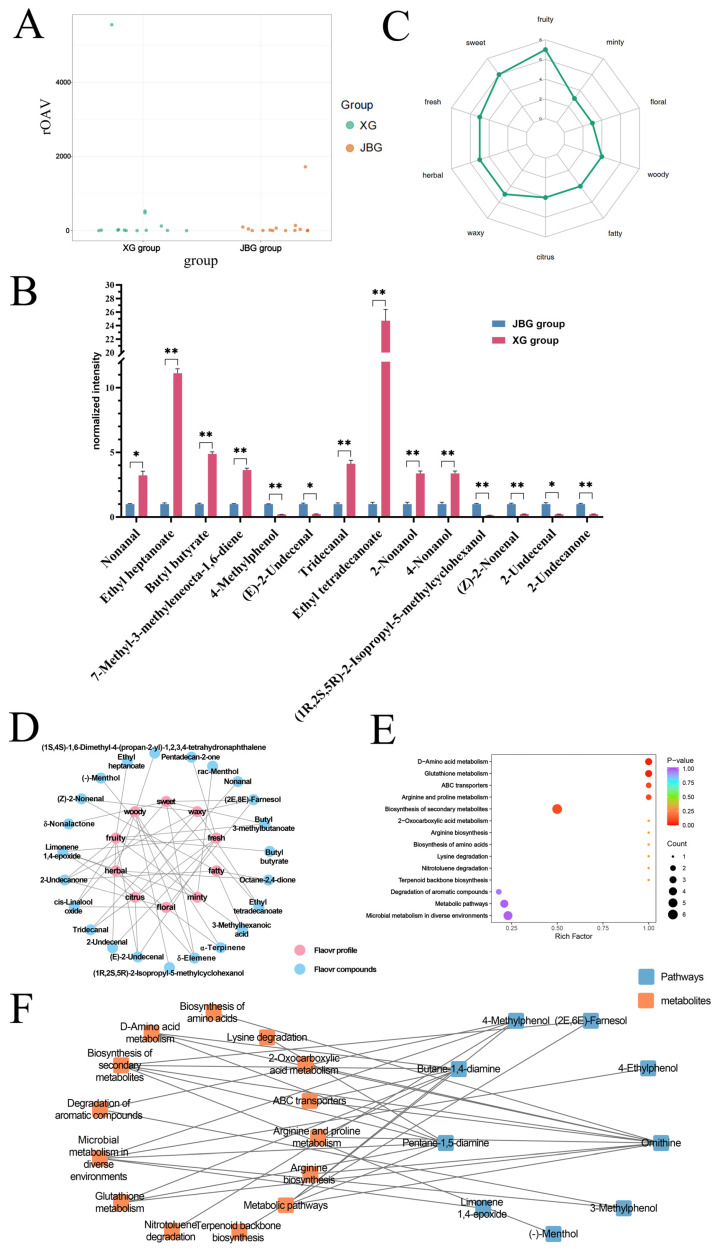
Functional annotation of volatile flavor compounds in LD muscles from Xizang goats and Jianzhou big-ear goats. (**A**) The scatter plot comparing compounds with relative Odor Activity Values (rOAV) ≥ 1 between the XG and JBG groups. (**B**) Radar chart of sensory aroma characteristics of differential VOCs between XG and JBG groups. (**C**) The relative intensities of 14 key VOCs between XG group and JBG groups. (**D**) Correlation network between sensory aroma characteristics and VOCs. The pink nodes represent flavor profile, and the blue nodes represent VOCs. (**E**) Differential abundant metabolite pathway enrichment maps. (**F**) Network diagram of the relationship between the significant pathway and differential metabolite analysis. The orange nodes on the left represent KEGG pathways, and the blue nodes on the right represent their corresponding differential abundant metabolites. * *p* < 0.05, ** *p* < 0.01.

The relative odor activity value (rOAV) is a widely used indicator for assessing the contribution of individual volatile compounds to overall flavor perception [18]. Using an established rOAV threshold of ≥1 [19], 68 VOCs were identified as major contributors to flavor, with odor descriptions summarized in Table S3. Through joint screening based on both differential abundance (|log_2_(fold change)| ≥ 1, *p* < 0.05 and VIP > 1) and rOAV ≥ 1), 14 key VOCs were identified as likely drivers of flavor differences. Among these, nonanal displayed the highest odor potency, with an rOAV exceeding 1700 (Figure 5A). We further displayed the concentrations of these 14 key VOCs between two groups. The levels of nonanal, ethyl heptanoate, butyl butyrate, 7-methyl-3-methyleneocta-1,6-diene, tridecanal, ethyl tetradecanoate, 2-nonanol and 4-nonanol were significantly upregulated in XG meat. For example, nonanal was approximately 3.23-fold higher in XG. In contrast, the concentrations of (1R, 2S, 5R)-2-Isopropyl-5-methylcyclohexanol, (Z)-2-nonenal, 2-undecenal, 2-undecanone, 4-methylphenol, and (E)-2-undecenal were significantly higher in JBG meat (Figure 5B).

The ten most frequently occurring flavor descriptors were fruity, sweet, fresh, herbal, waxy, citrus, fatty, woody, floral, and minty (Figure 5C). Among these, fruity was linked to the highest number of metabolites, indicating that fruity flavor attributes represent the primary sensory distinction between the XG and JBG meat samples (Figure 5C). To further investigate the relationship between sensory attributes and flavor compounds, a correlation network was constructed based on the ten most frequent flavor descriptors and their associated compounds (Figure 5D). Among the 14 key VOCs identified, the elevated levels of ethyl heptanoate and butyl butyrate in XG meat were associated with fruity flavor notes, directly contributing to a more intense fruity sensory profile. Nonanal is associated with citrus flavor, and 7-methyl-3-methyleneocta-1,6-diene mapped to the balsamic flavor characteristics (Table S3). These compounds collectively contributed to the richer, more layered flavor profile traditionally attributed to high-altitude XG meat. Conversely, the higher concentrations of (Z)-2-nonenal and 2-undecanone in JBG meat correlated with fatty flavor characteristics (Figure 5D).

#### 3.3.3. Analysis of Metabolite Pathway

To further investigate the potential metabolic pathways, the enrichment status of 86 differential metabolites was systematically evaluated via KEGG pathway analysis (Figure 5E). In total, 14 metabolic pathways, namely, biosynthesis of secondary metabolites, microbial metabolism in diverse environments, metabolic pathways, glutathione metabolism, D-amino acid metabolism, degradation of aromatic compounds, arginine and proline metabolism, 2−oxocarboxylic acid metabolism, arginine biosynthesis, biosynthesis of amino acids, lysine degradation, nitrotoluene degradation, terpenoid backbone biosynthesis, and ABC transporters, were enriched. Among them, the differential metabolites were the most enriched in the biosynthesis of secondary metabolite pathway, accounting for 60% of the total metabolites. The interaction network reveled the relationship between differential metabolites and enriched pathways (Figure 5F).

### 3.4. Correlational Analysis Between Lipids and VOCs and Flavor

**Figure 6:**
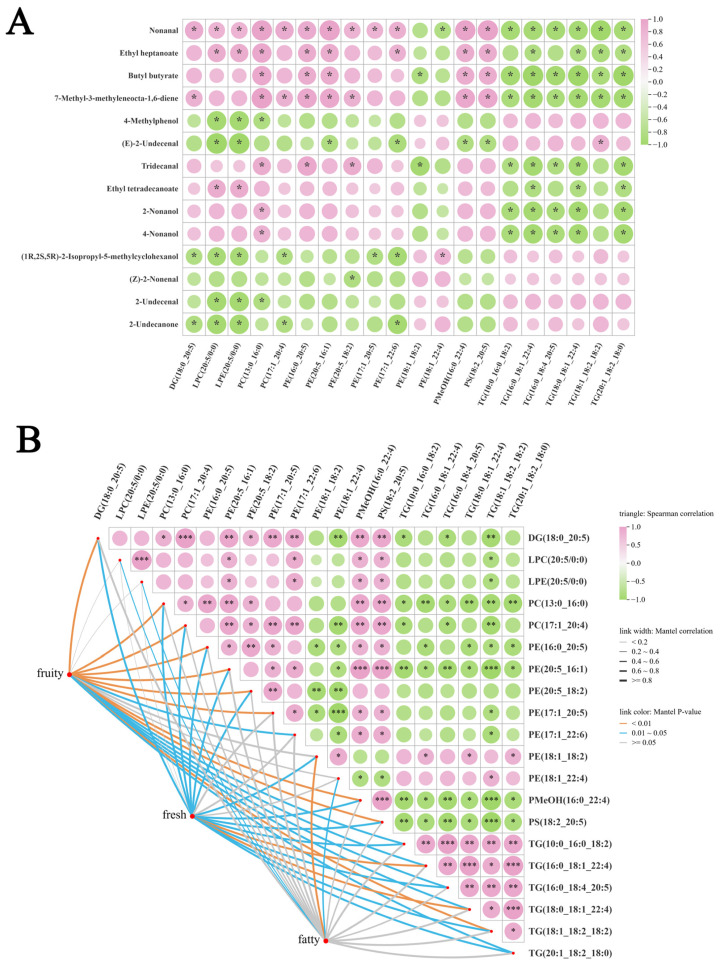
Correlational analysis between lipids and VOCs and flavor. (**A**) Spearman correlation analysis between the differential lipids and VOCs. Pink represents the positive correlation, and green represents the negative correlation. (**B**) Mantel’s correlation tests were used to determine the correlation between indicators with the key flavor and differential lipids between the XG and JBG groups. The thickness of the lines represents the correlation coefficient, with bold lines indicating Mantel’s r ≥ 0.8. The color of the lines represents significance, with orange indicating highly significant correlation (*p* < 0.01) and blue indicating significant correlation (0.01 < *p* < 0.05). Spearman’s test was used for correlation analysis between differential lipids, with pink indicating positive correlation, green indicating negative correlation. * *p* < 0.05, ** *p* < 0.01, *** *p* < 0.001.

The correlations between the 14 key VOCs and the 20 most significant lipids are illustrated in Figure 6A. The correlation heatmap revealed that nonanal, ethyl heptanoate, butyl butyrate, and 7-methyl-3-methyleneocta-1,6-diene exhibited positive correlations with most glycerophospholipids, including PC(13:0_16:0), PE(16:0_20:5), PE(20:5_16:1), PMeOH(16:0_22:4), and PS(18:2_20:5) (r > 0.8, *p* < 0.05). Tridecanal, 2-nonanol, and 4-nonanol were also positively correlated with PC(13:0_16:0). Conversely, VOCs abundant in JBG meat—such as 2-undecanone, 2-undecenal, (Z)-2-nonenal, and (1R, 2S, 5R)-2-Isopropyl-5-methylcyclohexanol—showed negative correlations with these glycerophospholipids. Instead, they were positively correlated with several triglycerides, including TG(10:0_16:0_18:2), TG(16:0_18:4_20:5), TG(18:0_18:1_22:4), and TG(18:1_18:2_18:2).

Mantel analysis was further employed to examine correlations between lipid profiles and the flavor attributes associated with the 14 key volatile compounds (Figure 6B). The results revealed significant positive correlations between fruity/fresh flavor attributes and specific glycerophospholipids, including PC(13:0_16:0), PC(17:1_20:4), PE(16:0_20:5), PE(20:5_16:1), PE(20:5_18:2), PE(17:1_20:5), PMeOH(16:0_22:4), and PS(18:2_20:5) (r > 0.4, *p* < 0.05). The distinct compositional differences in these lipid molecules between the two groups likely constitute a fundamental basis for the observed variations in fruity and fresh sensory notes. In contrast, fatty flavor was only correlated with four lipid species: LPC(20:5/0:0), LPE(20:5/0:0), PE(18:1_18:2), and PE(18:1_22:4).

## 4. Discussion

### 4.1. Lipids Analysis of Goats

Lipids play a crucial role in meat flavor development [35]. In the present study, the distinct lipid profiles observed between the XG and JBG groups, particularly the upregulation of specific glycerophospholipids in XG and the predominance of triglycerides in JBG, can be interpreted as an integrated outcome of genetic adaptation and environmental influence. This pattern reflects differential metabolic investments and physiological adaptations shaped by their respective high- and low-altitude habitats.

The observed lipidomic divergence likely stems from distinct metabolic priorities shaped by environmental pressures. For Xizang goats at high altitude, hypoxia is a predominant stressor. Cells sense hypoxia primarily through the stabilization of hypoxia-inducible factors (HIFs), which act as master transcriptional regulators [36]. The HIF pathway could upregulate the expression of enzymes involved in the biosynthesis of long-chain polyunsaturated fatty acids (PUFAs), such as arachidonic acid (20:4 n-6) and docosahexaenoic acid (22:6 n-3), and promote their incorporation into membrane glycerophospholipids like phosphatidylethanolamine and phosphatidylserine [37,38]. An increase in membrane PUFA content could enhance fluidity and flexibility, which is a crucial adaptation for maintaining cellular function under combined stresses of hypoxia and cold at high altitude [39,40]. Glycerophospholipids are integral components of goat meat and can significantly affect its quality and nutritional value [30]. In the XG group, the upregulation of glycerophospholipids such as PE(16:0_20:5), PE(20:5_16:1), PE(17:1_22:6), and PS(18:2_20:5) contained long-chain polyunsaturated fatty acids (PUFAs) like arachidonic acid (20:4, n-6) and docosahexaenoic acid (22:6, n-3). The releases of these free PUFAs could significantly affect the texture, flavor, and nutritional quality of meat and meat products [41]. Thus, the phospholipid-enriched profile in XG is not merely the integral components of goat meat but a direct metabolic signature of adaptation to high-altitude stress, involving enhanced fatty acid desaturation and phospholipid biosynthesis pathways [30,42].

In contrast, the JBG group displayed a lipid profile dominated by triglycerides. This reflects a metabolic physiology adapted to energy storage and rapid growth within its nutrient-rich, low-stress lowland habitat. Under such favorable conditions, the activity of key lipogenic enzymes was enhanced, promoting the de novo synthesis of fatty acids from dietary carbohydrates and their subsequent esterification into triglycerides for storage [43,44,45]. This metabolic shift is consistent with the breeding objectives for meat-type goats like JBG, which have been selected for traits such as fast growth and intramuscular fat (marbling) deposition [18,46]. The observed higher TG(10:0_16:0_18:2), TG(16:0_18:4_20:5), and TG(18:1_18:2_18:2), containing both medium- and long-chain fatty acids, aligns with a metabolic phenotype oriented toward efficient energy storage. Therefore, the TG-dominant lipidome in JBG reflects a breed-specific metabolic strategy optimized for meat production, facilitated by the energy-rich lowland environment.

### 4.2. Flavor Profile Analysis of Goats

Volatile flavor compounds are critical determinants of mutton aroma and taste [47]. In this study, ketones were the predominant volatile class in both goat groups. Ketones, characterized by their chemical stability and relatively high odor thresholds, contribute subtle yet foundational notes to the overall flavor profile [48]. Heterocyclic compounds further define the distinctive flavor of cooked meat [49]. Furans, such as 2-furfural, impart sweet, caramel-like aromas, while pyrroles (e.g., 2-acetylpyrrole) contribute meaty and smoky notes through Maillard reactions and amino acid degradation [1,50]. The significantly higher levels of heterocyclic compounds in XG meat suggested a greater availability of requisite precursors, such as free amino acids and reducing sugars, in its muscle post-mortem [49,51]. This may be linked to its specific metabolic state or proteolytic activity influenced by high-altitude adaptation. Esters, derived from lipid hydrolysis and oxidation during heating, enhance meat palatability with fruity and sweet notes [52]. Aldehydes—such as nonanal produced from oleic acid oxidation—impart rich, fatty, and sometimes green aromatic nuances that are central to meat flavor perception [1,20]. The elevated levels of esters and specific aldehydes in XG meat could be attributed to its phospholipid-enriched lipid profile. Phospholipids, particularly those rich in polyunsaturated fatty acids (PUFAs), serve as preferred substrates for the generation of these volatile compounds during thermal processing [53,54]. XG meat exhibited elevated levels of ketones, heterocyclic compounds, and esters, collectively contributing to a more layered and desirable flavor profile compared to JBG meat.

The distinct volatile profiles observed between XG and JBG meats also suggested fundamental differences in their flavor chemistry and underlying metabolism. In the present study, we identified 14 key VOCs as drivers of flavor differences between two groups. In XG meat, the significant upregulation of compounds such as nonanal, ethyl heptanoate, butyl butyrate, and 2-nonanol is particularly notable. Nonanal, derived mainly from lipid oxidation of ω-9 fatty acids, is known for its fatty, citrus-like, and roasted aromas, which are generally associated with desirable cooked meat notes [55,56]. Esters such as ethyl heptanoate and butyl butyrate, typically formed via esterification reactions between alcohols and carboxylic acids during post-mortem aging or heating [57], contribute distinct fruity and sweet nuances that enhance overall flavor complexity [58]. Conversely, JBG meat exhibited significantly elevated levels of (Z)-2-nonenal, (E)-2-undecenal, and 4-methylphenol. (Z)-2-nonenal and (E)-2-undecenal are typical secondary products of polyunsaturated fatty acid oxidation (especially n-6 fatty acids) and are often associated with fatty aroma [59]. Although the low concentrations of these compounds can contribute to the characteristic “meaty” aroma, their elevated presence may lead to a stronger, more penetrating or potentially off-flavors when present above certain thresholds [60]. The notable increase in 4-methylphenol is particularly indicative of differences in microbial metabolism, possibly linked to gastrointestinal microbiome variations between two groups [61]. This compound is a direct microbial metabolite of tyrosine fermentation [62]. Its higher concentration in JBG suggested that the gastrointestinal microbiome of this low-altitude breed provides a more favorable microenvironment for this metabolic activity [61,63,64]. Consequently, elevated 4-methylphenol could serve as a specific chemical signature reflecting these underlying digestive and metabolic disparities, contributing to the distinct volatile profile of JBG meat.

The distinct volatile profiles point to underlying biochemical differences between the two breeds. It is critical to contextualize the source of these detected volatile compounds. These VOCs were not merely released from the raw meat but were largely formed during thermal processing [32]. Thermal processing integrates a network of chemical reactions, including lipid oxidation, the Maillard reaction, and Strecker degradation [65]. The profile reported here thus represents the aroma-active end products of these thermally driven transformations, which collectively define the sensory profile of cooked meat.

### 4.3. Comprehensive Analysis of Goats

To this end, an integrated analysis of the data revealed a relationship between the raw meat lipid profiles and the cooking-generated flavor compounds, providing a biochemical basis for the observed sensory differences. Glycerophospholipids, especially PCs and PEs, were recognized as the primary substrates for lipid oxidation during cooking, generating aldehydes, ketones, and esters that define the desirable meaty, fruity, and complex aroma notes of cooked meat [66]. The specific correlation between fruity and fresh flavor attributes and PUFA-rich glycerophospholipids (e.g., PC(13:0_16:0), PE(20:5_16:1)) directly linked the unique phospholipid composition of XG muscle to its superior flavor profile [67]. Mechanistically, phospholipids, due to their localization in cell membranes in close proximity to heme iron and other pro-oxidants, are more susceptible to rapid oxidation during heating compared to triglycerides stored in lipid droplets [53]. The oxidation of PUFA-rich phospholipids could generate a diverse array of short- and medium-chain volatile carbonyls responsible for complex aromas [53,54]. Triglycerides, while also sources of fatty acids for oxidation, typically generate a different spectrum of volatiles, often associated with simpler, fattier notes [68]. The positive correlation between these volatiles and triglycerides reflected the breed’s metabolic emphasis on energy storage in low-altitude, fast-growing JBG [69]. This relationship can be explained by distinct oxidation pathways. Triglycerides, stored in lipid droplets, are enriched in longer-chain fatty acids [70]. Their oxidation, particularly during cooking, preferentially generates higher molecular weight aldehydes and ketones, such as the (Z)-2-nonenal and 2-undecenal identified in this study. These compounds are characteristic of a deeper, fattier aroma profile [59,68]. In contrast, the phospholipid-enriched membranes in XG muscle contain a higher proportion of polyunsaturated fatty acids situated near pro-oxidants. Their oxidation yields a broader spectrum of shorter-chain, highly volatile carbonyls (e.g., specific aldehydes and ketones), which contribute to the more complex and desirable flavor notes associated with Xizang goat meat [71]. Consequently, the overall flavor profile of JBG would be more strongly influenced by the oxidation products of its abundant stored triglycerides, whereas the profile of XG is shaped by its unique phospholipid composition. The Mantel analysis reinforces this mechanistic link, showing that the enrichment of specific phospholipid species in XG provides a direct biochemical substrate basis for its differentiated and often more desirable sensory attributes.

These findings are based on correlations that meaningfully connect raw meat lipid profiles to thermally treated meat flavor. Although lipid profiles were determined in raw tissue, VOCs and rOAVs were analyzed after cooking, the lipid precursors (notably glycerophospholipids and triglycerides) undergo oxidation to form the aldehydes, ketones, and esters that define aroma. Consequently, our model directly linked precursor composition to final sensory quality, offering practical insights for improving flavor through raw meat traits.

### 4.4. Limitations and Future Perspectives

These findings established the connection between lipid molecules and flavor in locally adapted goat breeds. Nevertheless, this study has certain limitations. Firstly, as the research focused primarily on molecular profiling, detailed carcass traits and physical meat quality measurements were not systematically collected. Future work integrating such phenotypic data with multi-omics approaches would help translate the identified metabolic markers into practical breeding or quality-assessment tools. Secondly, since the animals were reared in their respective native environments, the observed differences reflect an inherent breed × environment interaction, which complicates precise attribution of metabolic variations to either genetic or environmental factors alone. Future research can therefore prioritize disentangling these contributions through controlled experimental designs. Furthermore, expanding the multi-omics approach to integrate transcriptomics, proteomics, and metagenomics would illuminate the regulatory networks connecting genetic adaptation to lipid metabolism and flavor formation. Validating the identified lipid and volatile markers across larger, genetically diverse populations and correlating them with quantitative sensory analysis will enhance the applicability of the findings.

## 5. Conclusions

This study compared the muscle volatile flavor compounds and lipid profiles of the high-altitude Xizang goat (XG) and the low-altitude Jianzhou big-ear goat (JBG), revealing a fundamental metabolic divergence shaped by distinct environmental adaptations. Specifically, the phospholipid-enriched profile of XG, likely an adaptation to hypoxic stress, serves as a critical precursor pool for generating key fruity esters during thermal processing. In contrast, the triglyceride-dominated lipid profile of Jianzhou big-ear goat meat supports a simpler, fattier volatile spectrum characteristic of breeds adapted to energy-rich lowland environments. These findings provide a metabolite-level rationale for regional meat quality differences and highlight lipid metabolism as a central target for understanding and enhancing flavor in locally adapted goat breeds. Future research could focus on validating these lipid-flavor associations in larger populations and dissecting the genetic vs. environmental effects through controlled multi-omics experimental designs.

## Data Availability

Data will be made available on request.

## Supplementary Materials

The following supporting information can be downloaded at: https://www.mdpi.com/article/10.3390/foods15050855/s1, Table S1: Concentrate Composition and Nutrition Level (dry matter basis). Table S2: All VOCs identified in XG vs. JBG comparison. Table S3: 68 flavor-contributing VOCs and their odor descriptors. Figure S1: The total ion current peak detection results of quality control sample. (A) Represents the total ion current chromatogram in positive ion mode. (B) Represents the total ion current chromatogram in negative ion mode. Figure S2: The total ion current peak detection results of quality control sample.
