# Supplementary material for: Flavor Formation in Goat Meat: A Lipid-Centric Comparative Study of High-Altitude and Low-Altitude Breeds
Source: Foods. 2026 Mar 4;15(5):855. doi: 10.3390/foods15050855 (PMC12984371; doi:10.3390/foods15050855)
Supplement: Supplementary file 1 [file foods-15-00855-s001.zip › foods-4082250-supplementary.pdf]

Supplementary Table S1. Concentrate Composition and Nutrition Level (dry matter basis).

Item	Content	Nutritional level (Calculated)	
**Ingredients, %**			
Corn	58.50	Metabolizable energy, MJ/kg	11.80
Soybean meal	16.00	Crude protein, %	17.2
Wheat bran	15.00	Neutral detergent fiber, %	16.5
Rapeseed meal	7.00	Calcium, %	0.74
Limestone	1.20	Phosphorus, %	0.51
Salt	0.50		
Dicalcium Phosphate	0.60		
Premix [1]	1.00		
Extruded soybean	0.2		
Total	100.0		

[1] Premix provided the following for per kg of diets: VA 940 IU, VE 20 IU, S 200 mg, Fe 25 mg, Zn 40 mg, Cu 8 mg, I 0.3 mg, Mn 40 mg, Se 0.2 mg, Co 0.1 mg.

Supplementary Table S2. All VOCs identified in XG vs JBG comparison.

Index	Compound name	Kovats retention index	CAS	XG group	JBG group
AMW1754	3-Hexanol	780	623-37-0	1.55±0.17	1.39±0.04
AMW0885	1,2,3-trimethoxy-Propane	781	20637-49-4	0.10±0.02	0.10±0.01
AMW1843*045	1H-Pyrrole, 2-methyl-	799	636-41-9	0.03±0.01	0.01±0.00
AMW1722*045	3-methyl-1H-Pyrrole	799	616-43-3	0.03±0.01	0.01±0.00
AMW1798	2-Hexanol	799.79	626-93-7	0.22±0.18	0.06±0.04
AMW1656	Diallyl Sulfur compounds	860.18	592-88-1	5.95±0.94	4.94±0.04
AMW1054	3-ethyl-Cyclohexene	862	2808-71-1	0.16±0.03	0.13±0.00
AMW0151	Ethylbenzene	862.67	100-41-4	11.90±1.54	9.06±0.19
AMW0367	Piperidine	865	110-89-4	1.13±0.14	0.91±0.02
AMW2020	2,3-dimethyl-1,3-Heptadiene	868	74779-65-0	0.08±0.06	0.09±0.00
AMW0740	2-Methoxythiophene	870	16839-97-7	2.34±0.22	1.98±0.04
AMW0301*058	Benzene, 1,3-dimethyl-	870.58	108-38-3	5.58±1.41	3.63±0.02
AMW1265	ethenyl-Pyrazine	871	4177-16-6	4.99±3.72	5.83±0.05

AMW0242*058	p-Xylene	871.1	106-42-3	5.58±1.41	3.63±0.02
AMW1462	3-ethyl-Pyridine	887	536-78-7	0.45±0.11	0.29±0.01
AMW1819	1,3,5,7-Cyclooctatetraene	888	629-20-9	0.18±0.04	0.11±0.01
AMW1461	Phenylethyne	891	536-74-3	0.08±0.02	0.05±0.00
AMW2240*058	o-Xylene	894.21	95-47-6	5.58±1.41	3.63±0.02
AMW1153	Bicyclo[3.1.0]hexane, 4-methylene-1-(1-methylethyl)-	897	3387-41-5	0.02±0.00	0.01±0.00
AMW0845	5-methyl-Hexanenitrile	898	19424-34-1	0.28±0.02	0.10±0.01
AMW0666*055	2-ethyl-1H-Pyrrole	899	1551-06-0	0.04±0.01	0.02±0.00
AMW0667*055	3-ethyl-1H-Pyrrole	899	1551-16-2	0.04±0.01	0.02±0.00
AMW1065	2-methyl-5-propan-2-ylbicyclo[3.1.0]hex-2-ene	902	2867-05-2	3.59±2.49	3.80±0.05
AMW0358	Butane-1,4-diamine	905	110-60-1	0.26±0.01	0.09±0.01
AMW0389	Ethanol, 2-butoxy-	905.48	111-76-2	0.29±0.23	0.15±0.02
AMW2005*057	3,3,5-trimethyl-1,5-Heptadiene	906	74630-29-8	0.06±0.02	0.02±0.00
AMW1181*057	3,3,6-trimethyl-1,5-Heptadiene	906	35387-63-4	0.06±0.02	0.02±0.00
AMW1565	isothiocyanato-Cyclopropane	951	56601-42-4	0.06±0.04	0.06±0.00
AMW0983	Propanoic acid, 2-methyl-, 2-methylbutyl ester	955	2445-69-4	0.06±0.00	0.04±0.00
AMW2217	6-methyl-2-Heptanone	956	928-68-7	0.10±0.01	0.07±0.00
AMW1809	2-chloro-Octane	957	628-61-5	0.06±0.01	0.03±0.00
AMW1441	2,3,6,7-tetramethyl-Octane	958	52670-34-5	0.11±0.00	0.07±0.01
AMW1440	3-methyl-6-(1-methylethyl)-Cyclohexene	959	5256-65-5	0.02±0.01	0.02±0.00
AMW0727*067	3-Octen-2-one	960	1669-44-9	0.13±0.09	0.14±0.01
AMW0603*067	4-Octen-3-one	960	14129-48-7	0.13±0.09	0.14±0.01
AMW1528	.beta.-Phellandrene	964	555-10-2	0.04±0.00	0.02±0.01
AMW2126	N-methyl-Acetamide	964	79-16-3	0.09±0.01	0.07±0.00
AMW1329	2,6-Dimethyl-1,3,5,7-octatetraene, E,E-	966	460-01-5	0.21±0.07	0.15±0.01
AMW1769	Propanoic acid, pentyl ester	969	624-54-4	0.11±0.01	0.07±0.01
AMW0386	1-Heptanol	970.42	111-70-6	0.04±0.01	0.02±0.01
AMW0324	1-methyl-Piperazine	978	109-01-3	0.25±0.04	0.08±0.02
AMW1156	1-Octen-3-ol	979.76	3391-86-4	2.17±1.55	1.43±0.23

AMW1584	(1-methylethylidene)-Cyclohexane	980	5749-72-4	0.25±0.02	0.06±0.01
AMW1601*072	Octane-2,4-dione	984	585-25-1	19.36±1.02	4.06±0.51
AMW1129*072	2,5-Octanedione	984	3214-41-3	19.36±1.02	4.06±0.51
AMW2160	2-methyl-Butanoic acid,2-methyl-2-propenyl ester	986	83783-90-8	0.34±0.05	0.12±0.04
AMW0665	Cyclohexa-2,4-dienylmethanol	988	154916-94-6	0.22±0.03	0.08±0.01
AMW2247	Acetyl valeryl	989	96-04-8	2.14±1.53	1.39±0.22
AMW1278	Sorbic acid vinyl ester	990	42739-26-4	0.31±0.03	1.35±0.09
AMW0098	Isovaleric acid, 3-methylbutyl-2 ester	990	1000360-64-9	7.71±6.60	1.29±1.05
AMW1427	1-Tetrazol-2-ylethanone	991	51410-11-8	23.81±1.23	4.98±0.65
AMW0514	7-Methyl-3-methylideneocta-1,6-diene	992.02	123-35-3	1.88±0.13	0.52±0.03
AMW2243*084	Benzene, 1,2,4-trimethyl-	994.94	95-63-6	1.93±0.33	1.12±0.10
AMW1118	Ethanone, 1-(2-methyl-1-cyclopenten-1-yl)-	996	3168-90-9	0.07±0.01	0.03±0.00
AMW0328	Butyl butyrate	996.05	109-21-7	13.53±0.74	2.77±0.36
AMW1252	3-(2-methylpropyl)-Cyclohexene	997	4104-56-7	1.73±0.20	0.60±0.07
AMW1333	Pentane-1,5-diamine	1004	462-94-2	1.10±0.11	0.31±0.03
AMW1211	1-(3-methylbutyl)-Cyclopentene	1005	37689-15-9	0.43±0.04	0.10±0.01
AMW0825	cis-1-methyl-4-(1-methylethenyl)-Cyclohexane	1008	1879-07-8	0.38±0.03	0.10±0.01
AMW2227	2,5-Dimethylcyclohexanone	1013	932-51-4	0.04±0.00	0.14±0.01
AMW0763	Nonane, 2,6-dimethyl-	1018	17302-28-2	0.02±0.00	0.02±0.00
AMW1192	5-Ethylcyclopent-1-enecarboxAldehyde	1020	36431-60-4	0.03±0.02	0.03±0.00
AMW1094	6-methyl-2,4-Heptanedione	1024	3002-23-1	0.03±0.02	0.03±0.00
AMW1442*084	Benzene, 1,2,3-trimethyl-	1024.23	526-73-8	1.93±0.33	1.12±0.10
AMW0119	1-Hexanol, 2-ethyl-	1028.65	104-76-7	0.04±0.04	0.06±0.01
AMW0811	α-Terpinene	1029	18368-95-1	0.00±0.00	0.01±0.00
AMW0589	Pyrazine, 2-methyl-5-(1-methylethyl)-	1029	13925-05-8	0.04±0.00	0.04±0.00
AMW0475	Limonene 1,4-epoxide	1031	1195-92-2	0.07±0.01	0.26±0.02
AMW1677	D-Limonene	1031.27	5989-27-5	0.06±0.05	0.05±0.01
AMW0555	4-methyl-1-Decene	1041	13151-29-6	0.02±0.00	0.07±0.01

AMW1317	3-Methylhexanoic acid	1042.11	4536-23-6	0.09±0.10	0.20±0.04
AMW1747	2,2,4,4-Tetramethyloctane	1045	62183-79-3	0.27±0.19	0.11±0.12
AMW0849	2-Heptanone, 4,6-dimethyl-	1045	19549-80-5	1.29±0.86	3.65±0.69
AMW2196	2-hydroxy-BenzAldehyde	1045.6	90-02-8	0.05±0.04	0.02±0.00
AMW0502	BenzeneacetAldehyde	1045.6	122-78-1	0.24±0.11	0.30±0.05
AMW0761	Nonane, 4,5-dimethyl-	1046	17302-23-7	0.60±0.08	0.41±0.06
AMW0327	Butyl 3-methylbutanoate	1046.35	109-19-3	0.04±0.00	0.22±0.08
AMW1675	methyl-Thiourea	1048	598-52-7	0.03±0.00	0.16±0.08
AMW0273	2(3H)-Furanone, 5-ethenyldihydro-5-methyl-	1049	1073-11-6	0.74±0.40	0.51±0.06
AMW1308*089	4-Nonanone	1052	4485-09-0	0.71±0.04	0.52±0.03
AMW1931	2(3H)-Furanone, 5-ethyldihydro-	1054.86	695-06-7	0.33±0.05	0.23±0.03
AMW0955	2,7-Octadien-1-ol	1057	23578-51-0	0.07±0.01	0.05±0.00
AMW2006	(Z)-4-methyl-2-Decene	1059	74630-30-1	0.05±0.02	0.04±0.01
AMW2004	(Z)-8-methyl-2-Decene	1059	74630-25-4	0.68±0.18	0.69±0.09
AMW0402	2-Butoxyethyl acetate	1060	112-07-2	0.25±0.05	0.17±0.03
AMW2044	6-Propenylbicyclo[3.1.0]hexan-2-one	1061	75283-46-4	0.11±0.02	0.11±0.01
AMW1724	2-Pyrrolidinone	1065.9	616-45-5	0.14±0.01	0.12±0.01
AMW0728	2-Acetylcyclopentanone	1067	1670-46-8	0.05±0.03	0.06±0.00
AMW1017*092	(Z)-2-Octen-1-ol	1067	26001-58-1	82.11±12.64	72.25±3.71
AMW0925*092	2-Octen-1-ol	1067	22104-78-5	82.11±12.64	72.25±3.71
AMW0813*092	2-Octen-1-ol, (E)-	1067.62	18409-17-1	82.11±12.64	72.25±3.71
AMW0121	5-Methyl-4-hexene-1-yl acetate	1068	1000426-93-8	0.03±0.02	0.05±0.01
AMW1316	4-Ethylcyclohexanol	1068	4534-74-1	0.88±0.37	0.74±0.09
AMW1041	Azulene	1069	275-51-4	13.56±2.97	12.34±0.65
AMW0243*083	4-Methylphenol	1073.39	106-44-5	0.01±0.00	0.03±0.00
AMW0302*083	3-Methylphenol	1075.43	108-39-4	0.01±0.00	0.03±0.00
AMW1658*094	4-Nonanol	1078	5932-79-6	0.56±0.05	0.17±0.04
AMW0407	Formic acid, heptyl ester	1081	112-23-2	0.27±0.05	0.10±0.02
AMW0210	BenzAldehyde, 4-methyl-	1084.21	104-87-0	0.02±0.01	0.02±0.00
AMW0970	Cyclohexanone, 2,2,6-trimethyl-	1086	2408-37-9	2.84±0.75	2.29±0.49
AMW0163	Methenamine	1088	100-97-0	0.24±0.03	0.19±0.02

AMW0651*097	Mequinol	1090	150-76-5	1.35±0.17	1.64±0.11
AMW2197*097	2-methoxy-Phenol	1090.66	90-05-1	1.35±0.17	1.64±0.11
AMW1146	3-Hexen-1-ol, propanoate, (Z)-	1091	33467-74-2	0.47±0.10	0.40±0.01
AMW2146*089	2-Nonanone	1091.46	821-55-6	0.71±0.04	0.52±0.03
AMW0201	Benzene, n-butyl-	1092	104-51-8	0.11±0.03	0.13±0.01
AMW0099	6-methyl-7-Oxa-8-azabicyclo[4.2.1]non-8-ene	1092	1000362-10-7	1.94±0.24	1.55±0.11
AMW1903	2-Nonanone, 3-(hydroxymethyl)-	1093	67801-33-6	2.21±0.26	0.98±0.05
AMW0356	(S)-(+)-6-Methyl-1-octanol	1094	110453-78-6	1.82±0.56	1.61±0.08
AMW2151	2H-Pyran-2-one, tetrahydro-6-methyl-	1095.14	823-22-3	0.40±0.04	0.31±0.01
AMW0237	Ethyl heptanoate	1097.8	106-30-9	1.06±0.06	0.10±0.02
AMW1815*094	2-Nonanol	1099.2	628-99-9	0.56±0.05	0.17±0.04
AMW0952	2-methyl-2-Decene	1100	23381-92-2	4.33±0.57	4.37±0.25
AMW2106	Linalool	1100.59	78-70-6	6.40±0.69	2.94±0.42
AMW1183	Methanesulfonamide, 1-chloro-N,N-dimethyl-	1101	35427-68-0	0.86±0.12	0.68±0.04
AMW2090	3-Cyclopentyl-1-propanol	1102	767-05-5	0.70±0.10	0.56±0.04
AMW1952	3,6-Dimethyl-2,3,3a,4,5,7a-hexahydrobenzofuran	1103	70786-44-6	0.04±0.00	0.03±0.00
AMW2242	o-Toluidine	1105	95-53-4	0.32±0.03	0.26±0.02
AMW0536	Nonanal	1105.03	124-19-6	5.56±0.97	1.72±0.12
AMW0572*100	1,2-diethyl-Benzene	1106	135-01-3	0.02±0.01	0.01±0.00
AMW0608*100	1,3-diethyl-Benzene	1106	141-93-5	0.02±0.01	0.01±0.00
AMW0698	6-Methyl-3,5-heptadiene-2-one	1107	1604-28-0	5.34±0.70	4.24±0.30
AMW0464	2-Methylheptanoic Acid	1109	1188-02-9	14.04±2.14	11.33±0.96
AMW1000	2-methoxy-2-Octen-4-one	1113	24985-48-6	0.58±0.10	0.11±0.01
AMW2195*104	Phenol, 2-ethyl-	1114	90-00-6	0.00±0.00	0.01±0.00
AMW0789	Benzene, 2-ethyl-1,4-dimethyl-	1119	1758-88-9	0.67±0.08	0.50±0.01
AMW0696	Benzene, 1-methyl-4-(1-methylpropyl)-	1141	1595-16-0	0.02±0.01	0.02±0.00
AMW1691	(Z)-2-Nonenal	1148	60784-31-8	0.01±0.00	0.03±0.00

AMW0477*114	(2R-cis)-5-methyl-2-(1-methylethyl)-Cyclohexanone	1148	1196-31-2	0.78±0.51	0.44±0.08
AMW0203*114	Cyclohexanone, 5-methyl-2-(1-methylethyl)-	1148	10458-14-7	0.78±0.51	0.44±0.08
AMW0145	3-methyl-Undecane	1150	1002-43-3	0.11±0.01	0.10±0.01
AMW1341	2,4-dimethyl-1-(1-methylethyl)-Benzene	1155	4706-89-2	0.40±0.06	0.21±0.03
AMW0765	2,3-Dimethyldecane	1157	17312-44-6	0.01±0.00	0.01±0.01
AMW0958	2-Methylisoborneol	1161	2371-42-8	4.81±4.27	0.06±0.00
AMW2102	2-(hydroxymethyl)-2-methyl-1,3-Propanediol	1162	77-85-0	8.35±0.91	3.56±0.44
AMW0646*116	rac-Menthol	1164	1490-04-6	0.00±0.00	0.01±0.00
AMW1678	cis-Linalool oxide	1164	5989-33-3	0.00±0.00	0.02±0.00
AMW2190	(1R,2S,5R)-2-Isopropyl-5-methylcyclohexanol	1164	89-78-1	0.29±0.03	2.12±0.21
AMW0506*104	4-Ethylphenol	1165.4	123-07-9	0.00±0.00	0.01±0.00
AMW0460	tetraethyl-Urea	1166	1187-03-7	3.69±0.40	1.57±0.20
AMW0593	2-Ethylpiperazine	1171	13961-37-0	0.01±0.00	0.06±0.01
AMW0022	(E)-2-Butenoic acid, 2-(methylenecyclopropyl)prop-2-yl ester	1175	1000158-24-3	0.18±0.02	0.04±0.01
AMW0934*116	(-)-Menthol	1176.94	2216-51-5	0.00±0.00	0.01±0.00
AMW1282	pentyl-Cyclohexane	1178	4292-92-6	0.11±0.01	0.59±0.05
AMW1746	3-ethyl-2,7-dimethyl-Octane	1180	62183-55-5	5.11±0.43	2.44±0.29
AMW1794	Hexanoic acid, butyl ester	1189.39	626-82-4	0.39±0.08	0.22±0.02
AMW0199	Anethole	1190	104-46-1	0.05±0.06	0.08±0.00
AMW0778	Undecane, 5,7-dimethyl-	1190	17312-83-3	0.59±0.10	0.34±0.05
AMW1361	Thiophene, 2-pentyl-	1192	4861-58-9	9.03±1.62	6.90±0.43
AMW1710	3-Amino-5-pyrazolol	1194	6126-22-3	1.73±0.30	1.28±0.07
AMW0947*120	BenzAldehyde, 2-ethyl-	1195	22927-13-5	0.31±0.06	0.23±0.01
AMW1168*120	BenzAldehyde, 3-ethyl-	1195	34246-54-3	0.31±0.06	0.23±0.01
AMW2012*122	(Z)-4-methyl-4-Undecene	1199	74630-57-2	0.61±0.18	0.32±0.01
AMW1703*122	4-methyl-4-Undecene	1199	61142-40-3	0.61±0.18	0.32±0.01
AMW2159*122	6-methyl-5-Undecene	1199	83687-45-0	0.61±0.18	0.32±0.01

AMW1572	2-methyl-2-Undecene	1199	56888-88-1	10.73±1.85	8.06±0.49
AMW0472	Methyl salicylate	1200	119-36-8	0.02±0.01	0.02±0.00
AMW0887	6-Nonynoic acid, methyl ester	1200	20731-17-3	0.24±0.07	0.19±0.01
AMW0597	Estragole	1202.48	140-67-0	0.06±0.06	0.08±0.00
AMW0989	Pyrazine, 2-methoxy-3-(2-methylpropyl)-	1204	24683-00-9	87.97±15.27	67.86±3.98
AMW2281	Carveol	1206	99-48-9	0.11±0.03	0.09±0.01
AMW0758	Undecane, 4,7-dimethyl-	1207	17301-32-5	1.20±0.13	0.78±0.02
AMW0895	1-Cyclohexene-1-carboxAldehyde, 4-(1-methylethenyl)-	1207	2111-75-3	7.85±1.38	5.99±0.35
AMW0680	BenzAldehyde, 2,4-dimethyl-	1208	15764-16-6	0.28±0.04	0.23±0.01
AMW0416	Dodecane	1214	112-40-3	0.33±0.09	0.31±0.04
AMW0755	2,8-dimethyl-Undecane	1220	17301-25-6	0.16±0.03	0.13±0.02
AMW0772	Undecane, 4,4-dimethyl-	1229	17312-68-4	0.12±0.02	0.10±0.01
AMW0770*126	2,2-dimethyl-Undecane	1229	17312-64-0	0.14±0.03	0.11±0.01
AMW0773*126	5,5-dimethyl-Undecane	1229	17312-73-1	0.14±0.03	0.11±0.01
AMW0771*126	Decane, 3-ethyl-3-methyl-	1229	17312-66-2	0.14±0.03	0.11±0.01
AMW0775*126	Undecane, 6,6-dimethyl-	1229	17312-76-4	0.14±0.03	0.11±0.01
AMW0234*125	2,6-Octadien-1-ol, 3,7-dimethyl-, (Z)-	1230.47	106-25-2	2.82±0.37	2.42±0.18
AMW1752	1,4-Benzenedicarbonitrile	1236	623-26-7	0.01±0.00	0.01±0.00
AMW0004	trans,trans-1,7-Dimethylspiro[4.5]decane	1236	1000111-72-6	20.63±2.54	17.46±1.22
AMW1299	1,1'-ethylidenebis-Cyclopentane	1236	4413-21-2	3.45±0.34	2.94±0.19
AMW1885	2-(4-Bromobutyl)-furan	1237	66356-49-8	3.43±0.45	2.81±0.20
AMW0118	N-ethyl-2-Furancarboxamide	1241	1000407-23-8	2.34±0.31	2.12±0.15
AMW0876	2'-methyl-Propiophenone	1242	2040-14-4	0.23±0.03	0.20±0.02
AMW1136	2-Pentoxy-tetrahydropyran	1243	32767-70-7	3.92±0.53	3.48±0.21
AMW1067	Benzofuran, 4,7-dimethyl-	1244	28715-26-6	0.01±0.00	0.01±0.00
AMW0169	(1R,2S,4r)-4-((E)-prop-1-en-1-yl)cyclopentane-1,2-diol	1244	1010481-89-9	0.97±0.05	0.79±0.08
AMW2192	Pulegone	1244.49	89-82-7	1.04±0.17	0.54±0.06
AMW0498	4-(1-methylethyl)-Benzaldehyde	1246.39	122-03-2	0.07±0.02	0.04±0.00
AMW1264	2-phenoxy-1-Propanol	1247	4169-04-4	5.33±0.67	4.49±0.31

AMW0196	Benzeneacetic acid	1249	103-82-2	0.50±0.10	0.38±0.03
AMW2290*127	Acetophenone, 4'-hydroxy-	1250	99-93-4	0.50±0.06	0.41±0.03
AMW0495*127	Ethanone, 1-(3-hydroxyphenyl)-	1250	121-71-1	0.50±0.06	0.41±0.03
AMW0410	isothiocyanato-Cyclohexane	1251	1122-82-3	4.22±0.34	3.74±0.23
AMW0999	2-Decenal, (Z)-	1252	2497-25-8	1.54±0.20	1.38±0.09
AMW0228	Caprolactam	1253.7	105-60-2	0.40±0.06	0.35±0.02
AMW0697	3,4-dihydro-6-methyl-2H-Pyran	1255	16015-11-5	1.61±0.22	1.42±0.08
AMW1233	(3R,6S)-2,2,6-Trimethyl-6-vinyltetrahydro-2H-pyran-3-ol	1255	39028-58-5	1.96±0.23	1.64±0.11
AMW0233*125	Geraniol	1255.61	106-24-1	2.82±0.37	2.42±0.18
AMW1707	Dodecane, 4-methyl-	1259	6117-97-1	0.07±0.01	0.06±0.01
AMW0200	2(3H)-Furanone, 5-butyldihydro-	1261	104-50-7	0.55±0.10	0.49±0.07
AMW0282	hexyl-Benzene	1263.81	1077-16-3	0.00±0.00	0.01±0.00
AMW0411	1-Decanol	1271.53	112-30-1	0.54±0.10	0.22±0.04
AMW1149	6-UndecylAmine	1278	33788-00-0	0.00±0.00	0.02±0.00
AMW0028	2-Hydroxymethyl-2-methyl-pyrrolidine-1-carboxaldehyde	1278	1000189-82-1	0.33±0.06	0.19±0.02
AMW0006	threo-2,5-Dimethyl-2-(2-methyl-2-tetrahydrofuryl)tetrahydrofuran	1287	1000112-56-1	0.17±0.03	0.16±0.02
AMW1291	Nonane, 2,2,4,4,6,8,8-heptamethyl-	1294	4390-04-9	0.64±0.02	0.39±0.06
AMW0405	2-Undecanone	1294.65	112-12-9	0.01±0.00	0.03±0.00
AMW1945	Ornithine	1297	70-26-8	0.01±0.00	0.06±0.01
AMW0065	Oxalic acid, isobutyl neopentyl ester	1301	1000309-72-6	0.27±0.17	0.41±0.07
AMW0835	Ketone, methyl 2,4,5-trimethylpyrrol-3-yl	1303	19005-95-9	0.02±0.01	0.03±0.00
AMW1140	δ-Nonalactone	1304	3301-94-8	0.01±0.00	0.06±0.00
AMW0889	(1.alpha.,2.beta.,5.beta.)-5-methyl-2-(1-methylethyl)-Cyclohexanol, acetate	1304	20777-45-1	0.02±0.00	0.03±0.00
AMW2179	1,3-dimethyl-2,4(1H,3H)-Pyrimidinedione	1307	874-14-6	0.05±0.00	0.01±0.00
AMW0421	Undecanal	1308.44	112-44-7	0.07±0.01	0.03±0.01

AMW0987	2-Undecenal	1311	2463-77-6	0.01±0.00	0.07±0.01
AMW1456	(E)-2-Undecenal	1311	53448-07-0	0.01±0.00	0.05±0.01
AMW1580	3-Hydroxy-4-methylbenzAldehyde	1316	57295-30-4	0.01±0.01	0.01±0.00
AMW0007	Pyrimidine, 4-butyl-3,4-dihydro-5-methyl-	1316	1000115-50-2	0.02±0.00	0.02±0.00
AMW2014	2,7,10-trimethyl-Dodecane	1320	74645-98-0	0.02±0.00	0.02±0.00
AMW1228*135	Dodecane, 2,6,10-trimethyl-	1320	3891-98-3	0.02±0.00	0.01±0.00
AMW1114*135	Dodecane, 2,6,11-trimethyl-	1320	31295-56-4	0.02±0.00	0.01±0.00
AMW0997	6-Tridecene	1321	24949-38-0	0.11±0.03	0.09±0.01
AMW1131	4-tert-Butylcyclohexyl acetate	1322	32210-23-4	0.56±0.11	0.50±0.07
AMW1143	2-pentyl-Piperidine	1324	33354-97-1	0.13±0.03	0.12±0.02
AMW0528	Hexanedioic Acid	1330	124-04-9	0.01±0.00	0.01±0.00
AMW0884	5-hydroxy-2,4(1H,3H)-Pyrimidinedione	1330	20636-41-3	0.03±0.01	0.02±0.00
AMW2099	Propanoic acid, 2-methyl-, 3-hydroxy-2,2,4-trimethylpentyl ester	1331	77-68-9	0.72±0.10	0.64±0.10
AMW1668	3-methyl-3-phenyl-Azetidine	1334	5961-33-1	0.06±0.04	0.04±0.01
AMW0534	2-(2-butoxyethoxy)-Ethanol,acetate	1334	124-17-4	0.17±0.11	0.09±0.02
AMW2202	Quinoline, 6-methyl-	1338	91-62-3	0.01±0.01	0.00±0.00
AMW1363	2-Cyclopenten-1-one, 3-methyl-2-(2-pentenyl)-, (Z)-	1338	488-10-8	0.02±0.01	0.02±0.00
AMW0352	2-methyl-Undecanal	1338	110-41-8	0.06±0.02	0.04±0.00
AMW0814	Pyrazine, 2,5-dimethyl-3-(3-methylbutyl)-	1341	18433-98-2	0.03±0.01	0.02±0.00
AMW0798	.alpha.-Cubebene	1344	17699-14-8	0.01±0.00	0.01±0.00
AMW0663	3-Dodecanone	1350	1534-27-6	0.01±0.00	0.01±0.00
AMW0734	3,5-Heptanedione, 4-ethyl-2,2,6,6-tetramethyl-	1352	167545-33-7	0.04±0.00	0.04±0.00
AMW1263	trans-3,5,6,8a-tetrahydro-2,5,5,8a-tetramethyl-2H-1-Benzopyran	1352	41678-29-9	0.05±0.02	0.05±0.00
AMW1693	2,3-dichloro-Benzenamine	1352	608-27-5	0.22±0.09	0.16±0.01
AMW0128	3-Ethyl-2,6,10-trimethylundecane	1355	1000432-25-9	0.14±0.02	0.13±0.01
AMW1412	Sarcosine anhydride	1361	5076-82-4	0.12±0.03	0.08±0.00
AMW0826	4-Picoline, 2-(tert-butylthio)-	1365	18794-36-0	0.02±0.00	0.02±0.00

AMW0600	2,6-Octadien-1-ol, 3,7-dimethyl-, acetate, (Z)-	1365.22	141-12-8	0.02±0.01	0.01±0.01
AMW0204	2(3H)-Furanone, dihydro-5-pentyl-	1368.03	104-61-0	0.20±0.23	0.19±0.12
AMW0871	δ-Elemene	1377	20307-84-0	0.02±0.02	0.06±0.02
AMW1650	Benzeneethanethioic acid, S-methyl ester	1379	5925-74-6	0.06±0.01	0.02±0.02
AMW0166	2-(4-bromobutyl)-5,5-dimethyl-1,3-Cyclopentadiene	1382	1010163-68-7	0.23±0.03	0.17±0.02
AMW0140	3-Methyl-1-adamantanecarboxylic acid	1384	1000504-38-9	0.27±0.04	0.23±0.02
AMW1689	6,8-Nonadien-2-one, 6-methyl-5-(1-methylethylidene)-	1387	60714-16-1	0.08±0.00	0.07±0.01
AMW0286	heptyl-Benzene	1390	1078-71-3	0.01±0.00	0.01±0.00
AMW0216	Betazole	1390	105-20-4	0.02±0.01	0.01±0.00
AMW0399	1-Tetradecene	1392	1120-36-1	0.05±0.01	0.03±0.01
AMW1517	2-(formyloxy)-1-phenyl-Ethanone	1393	55153-12-3	0.09±0.01	0.08±0.00
AMW1300	1H-Tetrazol-5-amine	1396	4418-61-5	0.94±0.17	0.48±0.03
AMW1822	Tetradecane	1400	629-59-4	1.58±0.33	0.76±0.05
AMW0425	Dodecanal	1402	112-54-9	0.12±0.01	0.08±0.00
AMW1876	1H-3a,7-Methanoazulene, 2,3,4,7,8,8a-hexahydro-3,6,8,8-tetramethyl-, (3R,3aS,7S,8aR)-	1403	65354-33-8	0.06±0.00	0.05±0.01
AMW0846	1,5-Cyclododecadiene, (E,Z)-	1403	19428-99-0	0.25±0.07	0.12±0.01
AMW0695	1-Methyl-2-n-hexylbenzene	1404	1595-10-4	0.04±0.01	0.03±0.01
AMW0034	Methyl 5(3)-methyl-4-hydroxy-3(5)-pyrazolecarboxylate	1406	1000211-81-5	0.02±0.00	0.02±0.00
AMW1237	2-Furancarboxylic acid, hexyl ester	1406	39251-86-0	0.65±1.10	0.19±0.24
AMW0174	Diphenyl ether	1409.71	101-84-8	0.01±0.00	0.01±0.00
AMW1269	1-(1-methylethyl)-2-nonyl-Cyclopropane	1412	41977-39-3	0.17±0.03	0.12±0.01
AMW1032	N1-(4-fluorobenzyl)-N2,N2-dimethyl-1,2-ethanediamine	1414	2714-80-9	0.11±0.01	0.06±0.03
AMW1325	[1R-(1.alpha.,3a.alpha.,7a.alpha.)]-1,2,3,6,7,7a-hexahydro-	1416	4545-68-0	0.01±0.00	0.01±0.00

	2,2,4,7a-tetramethyl-1,3a-Ethano-3aH-indene				
AMW0913	(E,E)-2,4-Dodecadienal	1418	21662-16-8	0.48±0.36	0.14±0.03
AMW0623	Azacyclohexane, 3-[1-pyrrolidyl]-	1419	144243-28-7	0.10±0.01	0.06±0.01
AMW1229	2,6,10-Trimethyltridecane	1419	3891-99-4	0.48±0.09	0.44±0.09
AMW0751	2(4H)-Benzofuranone, 5,6,7,7a-tetrahydro-4,4,7a-trimethyl-, (R)-	1426	17092-92-1	0.02±0.00	0.02±0.00
AMW0599	3,5,9-Undecatrien-2-one, 6,10-dimethyl-	1428	141-10-6	0.07±0.03	0.07±0.01
AMW0881	1-iodo-Decane	1430	2050-77-3	0.36±0.07	0.34±0.07
AMW0546	.alpha.-Ionone	1435.12	127-41-3	0.73±0.19	0.40±0.05
AMW1808	3,4-bis(1,1-dimethylethyl)-2,2,5,5-tetramethyl-Hexane	1440	62850-21-9	0.16±0.02	0.14±0.03
AMW0559	2-Bromo dodecane	1446	13187-99-0	0.27±0.06	0.25±0.05
AMW0815	3-methyl-Tetradecane	1448	18435-22-8	0.03±0.01	0.02±0.01
AMW0753	2-Butanone, 4-(2,6,6-trimethyl-1-cyclohexen-1-yl)-	1449	17283-81-7	0.05±0.01	0.02±0.01
AMW1066	13-Oxabicyclo[10.1.0]tridecane	1450	286-99-7	0.22±0.09	0.16±0.04
AMW1915	5,9-Undecadien-2-one, 6,10-dimethyl-	1452	689-67-8	0.08±0.01	0.03±0.00
AMW1217	5,9-Undecadien-2-one, 6,10-dimethyl-, (E)-	1453	3796-70-1	0.09±0.02	0.03±0.00
AMW0657	2-cyclopentyl-Phenol	1455	1518-84-9	0.02±0.00	0.01±0.00
AMW1072	Cyclopentane, nonyl-	1456	2882-98-6	0.22±0.18	0.04±0.01
AMW0424	1-Dodecanol	1457	112-53-8	0.17±0.04	0.16±0.04
AMW1177*142	2-Buten-1-one, 1-(2,6,6-trimethyl-1-cyclohexen-1-yl)-	1457	35044-68-9	76.29±18.60	35.08±5.01
AMW0647*142	β-Ionone	1457	14901-07-6	76.29±18.60	35.08±5.01
AMW1426	BenzeneacetAldehyde, .alpha.-methyl-4-(2-methylpropyl)-	1464	51407-46-6	0.01±0.02	0.01±0.00
AMW1918	Z-2-Dodecenol	1465	69064-36-4	0.83±0.17	0.71±0.15
AMW0011	2,10-dimethyl-9-Undecenol	1477	1000131-86-0	8.03±0.97	6.23±0.66
AMW2157	1,8-Ethylenenaphthalene	1478	83-32-9	0.38±0.04	0.41±0.05
AMW2084	Vinyl lauryl ether	1479	765-14-0	1.26±0.18	1.01±0.06
AMW1399	6-Methyl-6-(5-methylfuran-2-yl)heptan-2-one	1479	50464-95-4	6.85±1.26	3.22±0.24

AMW0088	D-Alanine, N-propargyloxycarbonyl-, propargyl ester	1480	1000347-74-5	2.74±0.11	2.47±0.23
AMW0391	Dodecanoic acid, methyl ester	1481	111-82-0	0.83±0.06	0.87±0.11
AMW0483	4-(2,6,6-Trimethylcyclohexa-1,3-dienyl)but-3-en-2-one	1485	1203-08-3	0.69±0.23	0.35±0.04
AMW2133*177	4-(2,2,3-trimethyl-6-methylenecyclohexyl)-3-Buten-2-one	1485	79-68-5	9.26±1.39	7.13±0.77
AMW0582	6,7-Dodecanedione	1486	13757-90-9	0.43±0.05	0.42±0.05
AMW1198	N-Ethyl-4-nitroaniline	1487	3665-80-3	1.59±0.25	1.50±0.12
AMW2134*177	.alpha.-Irone	1490	79-69-6	9.26±1.39	7.13±0.77
AMW0224	N-propyl-Benzamide	1492	10546-70-0	10.06±1.16	8.16±0.87
AMW2135*142	trans-.beta.-Ionone	1492.1	79-77-6	76.29±18.60	35.08±5.01
AMW0191	Propanoic acid, 2-methyl-, 3-phenylpropyl ester	1493	103-58-2	1.35±0.17	1.11±0.12
AMW1909	Benzaldehyde, 4-pentyl-	1493	6853-57-2	15.27±1.99	12.05±1.27
AMW0459	Bicyclo[7.2.0]undec-4-ene, 4,11,11-trimethyl-8-methylene-,[1R-(1R*,4Z,9S*)]-	1494	118-65-0	3.17±0.42	2.44±0.31
AMW1657	2-Tridecanone	1495.82	593-08-8	0.11±0.04	0.07±0.02
AMW1089	1-octenyl-Benzene	1497	29518-72-7	0.89±0.11	0.74±0.07
AMW0583	Hexathiane	1500	13798-23-7	4.57±1.12	2.07±0.31
AMW0718	2-Tridecanol	1510	1653-31-2	0.38±0.05	0.10±0.02
AMW0209	Tridecanal	1513	10486-19-8	1.02±0.12	0.25±0.04
AMW0063	Oxalic acid, cyclohexylmethyl ethyl ester	1513	1000309-68-0	0.55±0.07	0.08±0.08
AMW0108	Carbonic acid, nonyl prop-1-en-2-yl ester	1524	1000382-53-9	0.02±0.00	0.03±0.01
AMW1279	1-Iodoundecane	1529	4282-44-4	0.19±0.04	0.18±0.05
AMW1356	(1S,4S)-1,6-Dimethyl-4-(propan-2-yl)-1,2,3,4-tetrahydronaphthalene	1537	483-77-2	0.01±0.01	0.05±0.00
AMW1071	3-methyl-Pentadecane	1548	2882-96-4	0.02±0.00	0.02±0.00
AMW1053	Pentadecane, 4-methyl-	1548	2801-87-8	0.30±0.06	0.26±0.09
AMW0492	Vanillic acid	1560	121-34-6	0.03±0.00	0.03±0.00
AMW2027	(E)-2-Tridecen-1-ol	1564	74962-98-4	0.26±0.04	0.31±0.05

AMW0205	2(3H)-Furanone, 5-heptyldihydro-	1577	104-67-6	0.21±0.02	0.23±0.01
AMW0949	.gamma.-Dodecalactone	1582	2305-05-7	0.01±0.00	0.01±0.00
AMW1825	Cetene	1592	629-73-2	0.04±0.03	0.04±0.00
AMW0962	1H-Pyrazolo[3,4-d]pyrimidin-4-amine	1595	2380-63-4	0.03±0.01	0.01±0.00
AMW0167	Cyclohexanecarboxylic acid, 3-fluorophenyl ester	1596	1010330-87-9	0.03±0.00	0.01±0.00
AMW1963	.delta.-Dodecalactone	1602	713-95-1	0.18±0.02	0.08±0.01
AMW0476	Benzophenone	1603	119-61-9	0.07±0.00	0.03±0.00
AMW1499	Hexadecane	1612	544-76-3	0.13±0.01	0.06±0.02
AMW1281	Dodecane, 1-iodo-	1628	4292-19-7	0.05±0.01	0.04±0.01
AMW0500	Diphenylamine	1634.64	122-39-4	0.08±0.01	0.05±0.01
AMW0670	Hexadecane, 2-methyl-	1647	1560-92-5	0.85±0.07	0.35±0.12
AMW1432	(Z,E)-9,12-Tetradecadien-1-ol	1672	51937-00-9	0.14±0.02	0.02±0.00
AMW0062	Oxalic acid, ethyl 2-isopropylphenyl ester	1674	1000309-56-0	0.06±0.01	0.02±0.01
AMW1088	Cyclotetradecane	1679	295-17-0	0.06±0.01	0.02±0.00
AMW0080	Carbonic acid, butyl 4-isopropylphenyl ester	1682	1000331-42-0	0.07±0.00	0.02±0.01
AMW0165*145	cis-2,9-anti-9,10-cis-1,10-Tricyclo[8.6.0.0(2,9)]hexadeca-3,15-diene	1687	1010150-40-2	0.10±0.01	0.03±0.01
AMW0017*145	trans-2,9-anti-9,10-trans-1,10-Tricyclo[8.6.0.0(2,9)]hexadeca-3,15-diene	1687	1000150-40-1	0.10±0.01	0.03±0.01
AMW0954	Pentadecan-2-one	1698.33	2345-28-0	5.53±0.16	0.69±0.12
AMW1957	trans-3-Oxooctahydro-4a(2H)-naphthalenecarboxylic acid	1702	71198-51-1	0.75±0.06	0.17±0.02
AMW0069	d-Proline, N-methoxycarbonyl-, pentyl ester	1705	1000320-78-8	0.01±0.01	0.04±0.01
AMW0055	Sulfurous acid, 2-ethylhexyl isobutyl ester	1709	1000309-18-7	0.98±0.24	0.49±0.08
AMW0236	(2E,8E)-Farnesol	1710	106-28-5	0.10±0.01	0.03±0.01
AMW0072	4-(2-Ethylhexoxy)ethylbenzene	1714	1000327-15-7	0.11±0.01	0.04±0.01
AMW1014	8-Heptadecene	1719	2579-04-6	4.61±0.35	0.89±0.13
AMW0739	Di(n-hexyl)sulfone	1721	16823-61-3	0.05±0.00	0.21±0.03
AMW0207	1,8,11,14-Heptadecatetraene, (Z,Z,Z)-	1725	10482-53-8	0.39±0.05	0.08±0.02

AMW1542	Cyclopentanone, 3,3,4-trimethyl-4-(4-methylphenyl)-	1727	56077-23-7	0.04±0.01	0.02±0.00
AMW1036*149	(1-butyloctyl)-Benzene	1730	2719-63-3	0.04±0.01	0.02±0.00
AMW1034*149	(1-methylundecyl)-Benzene	1730	2719-61-1	0.04±0.01	0.02±0.00
AMW1035*149	(1-pentylheptyl)-Benzene	1730	2719-62-2	0.04±0.01	0.02±0.00
AMW0049	2-Methyl-furan-3-carboxylic acid N'-acetyl-hydrazide	1731	1000295-95-6	0.90±0.10	0.13±0.03
AMW0486	Benzyl Benzoate	1733	120-51-4	0.05±0.02	0.02±0.01
AMW1595	Myristic acid vinyl ester	1769	5809-91-6	0.01±0.00	0.04±0.01
AMW0529	Ethyl tetradecanoate	1779	124-06-1	2.46±0.29	0.10±0.02
AMW1961	Pentadecanoic acid, methyl ester	1779	7132-64-1	0.43±0.05	0.02±0.00
AMW1826	n-Pentadecanol	1779.19	629-76-5	0.13±0.05	0.06±0.01
AMW1664	Kaur-15-ene	1794	5947-50-2	0.00±0.00	0.01±0.00
AMW1660	Octadecane	1799.12	593-45-3	0.04±0.01	0.02±0.00
AMW1512	2-methyl-Hexadecanal	1835	55019-46-0	0.03±0.00	0.01±0.00
AMW0054	Sulfurous acid, butyl octyl ester	1837	1000309-17-5	0.21±0.03	0.08±0.00
AMW2237	2-phenyl-1H-Indole	1838	948-65-2	0.02±0.03	0.04±0.00
AMW1585	N-1-naphthalenyl-Acetamide	1844	575-36-0	0.00±0.00	0.01±0.00
AMW0116	Decyl octyl ether	1886	1000406-38-3	0.10±0.01	0.02±0.01
AMW0056	Sulfurous acid, 2-ethylhexyl isohexyl ester	1908	1000309-19-0	0.49±0.11	0.16±0.03
AMW0003	1-Meyhyl-2-heptyl-5-(5-hexenyl)pyrrolidine	1918	1000111-46-4	0.04±0.00	0.01±0.00

Data are means ± SEM (n=6)

Supplementary Table S3. 68 flavor-contributing VOCs and their odor descriptors.

Compounds	Odor
(1R,2S,5R)-2-Isopropyl-5-methylcyclohexanol	pepperminty, cool, woody
4-Methylphenol	phenol, narcissus, animalic, mimosa
(E)-2-Undecenal	fresh, fruity, citrus, orange, peel
(Z)-2-Nonenal	orris, fatty, waxy, cucumber
2-Nonanol	rose
2-Undecanone	waxy, fruity, creamy, fatty, orris, floral
2-Undecenal	fresh, fruity, orange, peel

7-Methyl-3-methylideneocta-1,6-diene	musty, balsamic, spice
Butyl 3-methylbutanoate	apple, pear, fruity, sweet, pineapple, green, peach
Butyl butyrate	fruity, banana, pineapple, green, cherry, tropical fruit, ripe fruit, juicy fruity
Ethyl heptanoate	fruity, pineapple, cognac, rummy, wine
Ethyl tetradecanoate	sweet, waxy, violet, orris
Limonene 1,4-epoxide	fresh, citrus, minty, spearmint, herbal
Nonanal	aldehyde, citrus, orange peel
Tridecanal	fresh, clean, aldehydic, soapy, citrus, petal, waxy, grapefruit, peel
.delta.-Dodecalactone	peachy, oily, creamy, soapy
2(3H)-Furanone, 5-heptyldihydro-	fruity, peach, creamy, fatty, lactonic, apricot, ketonic, coconut
2(3H)-Furanone, 5-butyldihydro-	sweet, coconut, waxy, creamy, tonka, dairy, fatty
2(3H)-Furanone, dihydro-5-pentyl-	coconut, woody
.gamma.-Dodecalactone	fatty, peachy, musky
2(3H)-Furanone, 5-ethyldihydro-	sweet, caramel
.beta.-Phellandrene	terpenic, herbal
β-Ionone	floral, woody, sweet, fruity, berry, tropical, beeswax
trans-.beta.-Ionone	dry, powdery, floral, woody, orris
.alpha.-Ionone	sweet, woody, floral, violet, orris, tropical, fruity
Pulegone	pepperminty, camphor, fresh, herbal, buchu
Propanoic acid, pentyl ester	sweet, fruity, apricot, pineapple, tropical, banana
Octadecane	alkane
2,6-Octadien-1-ol, 3,7-dimethyl-, (Z)-	lemon, fresh
Dodecanoic acid, methyl ester	waxy, soapy, creamy, coconut, mushroom
Cyclohexanone, 5-methyl-2-(1-methylethyl)-	minty
Linalool	floral, green
Geraniol	sweet, floral, fruity, rose, waxy, citrus
Estragole	sweet, sassafrass, anisic, spice, green, herbal, fennel
D-Limonene	citrus
Diallyl Sulfur compounds	sulfury, onion, garlic, horseradish, metallic
2-Cyclopenten-1-one, 3-methyl-2-(2-pentenyl)-, (Z)-	woody, herbal, floral, spicy, jasmin, celery
Benzeneacetic acid	sweet, honey, floral, honeysuckle, sour, waxy, civet
BenzeneacetAldehyde	floral, honey, rose, cherry

2H-Pyran-2-one, tetrahydro-6-methyl-	creamy, fruity, coconut
6-methyl-2-Heptanone	camphor
4-Octen-3-one	coconut, fruity
1-Cyclohexene-1-carboxAldehyde, 4-(1-methylethenyl)-	fresh, green, herbal, grassy, sweet, minty, cumin
2-Butanone, 4-(2,6,6-trimethyl-1-cyclohexen-1-yl)-	earthy, woody, mahogany, orris, dry, amber
3-Octen-2-one	earthy, spicy, herbal, sweet, mushroom, hay, blueberry
3-Hexanol	alcoholic, fusel, rummy, whiskey, guava, apple
3,6-Dimethyl-2,3,3a,4,5,7a-hexahydrobenzofuran	herbal, dill
2-Octen-1-ol	green, vegetable
2-Nonanone	fruity, floral, fatty
2-Methylisoborneol	earthy, musty
Propanoic acid, 2-methyl-, 2-methylbutyl ester	fruity, ethereal, tropical, banana
2-methoxy-Phenol	nutty
Pyrazine, 2-methoxy-3-(2-methylpropyl)-	green bell pepper, pea, galbanum
ethenyl-Pyrazine	green, burnt, nutty
Cyclohexanone, 2,2,6-trimethyl-	pungent, thujone, labdanum, honey, cistus
1-Octen-3-ol	fatty, fruity, grassy, mushroom, perfumy, sweet
1-Heptanol	grassy
1-Decanol	fatty, waxy, floral, orange, sweet, watery
p-Xylene	strong, sweetish
Benzene, 1,2,4-trimethyl-	plastic
Benzene, 1,2,4-trimethyl-	plastic
(Z)-2-Octen-1-ol	sweet, floral
2-Decenal, (Z)-	tallow
Anethole	sweet, exotic, flowery, stewed
5,9-Undecadien-2-one, 6,10-dimethyl-, (E)-	fresh, green, fruity, waxy, rose, woody, magnolia, tropical
2-Octen-1-ol, (E)-	green, citrus, vegetable, fatty
Cyclohexanone, 5-methyl-2-(1-methylethyl)-	minty
2(4H)-Benzofuranone, 5,6,7,7a-tetrahydro-4,4,7a-trimethyl-, (R)-	musky, coumarin

Supplementary Figure S1.

A

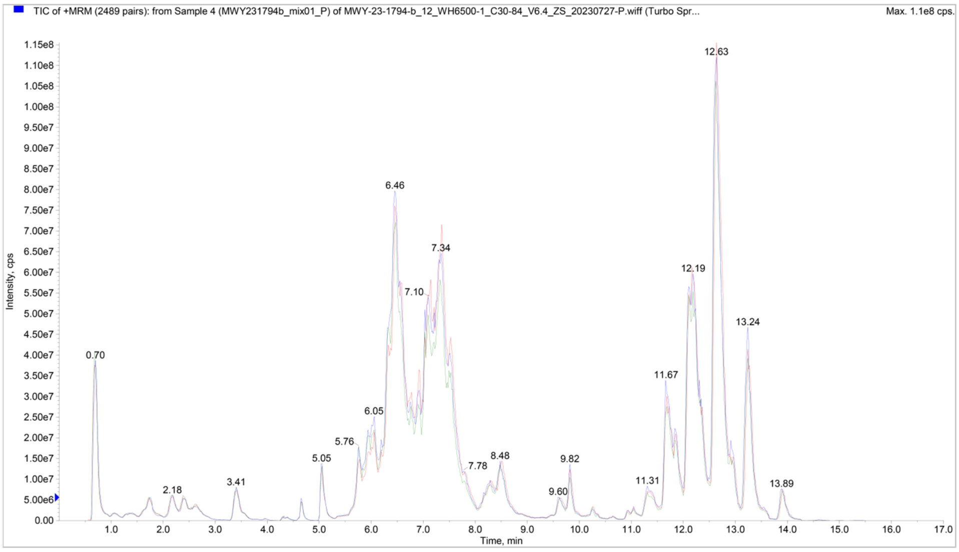

B

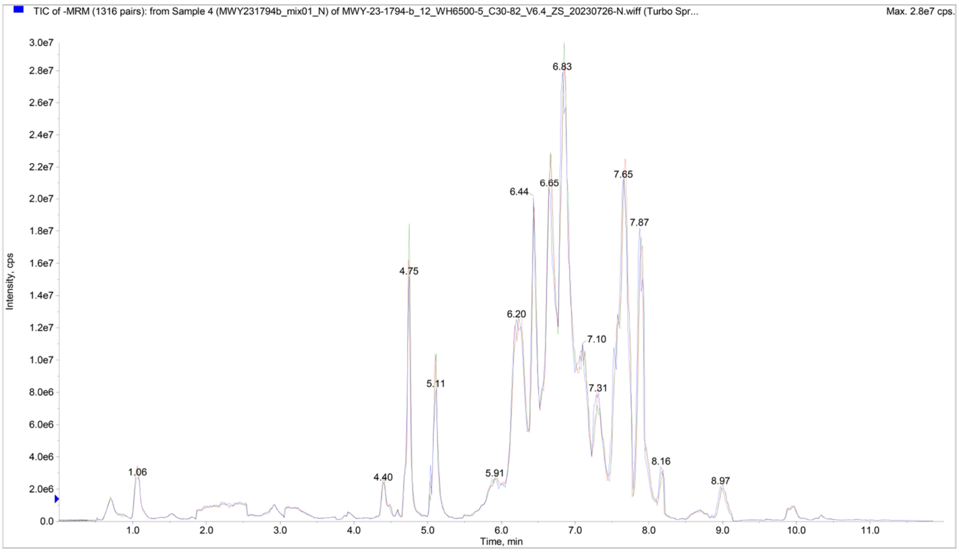

The total ion current peak detection results of quality control sample. (A) The total ion current chromatogram in positive ion mode. (B) The total ion current chromatogram in negative ion mode.

Supplementary Figure S2.

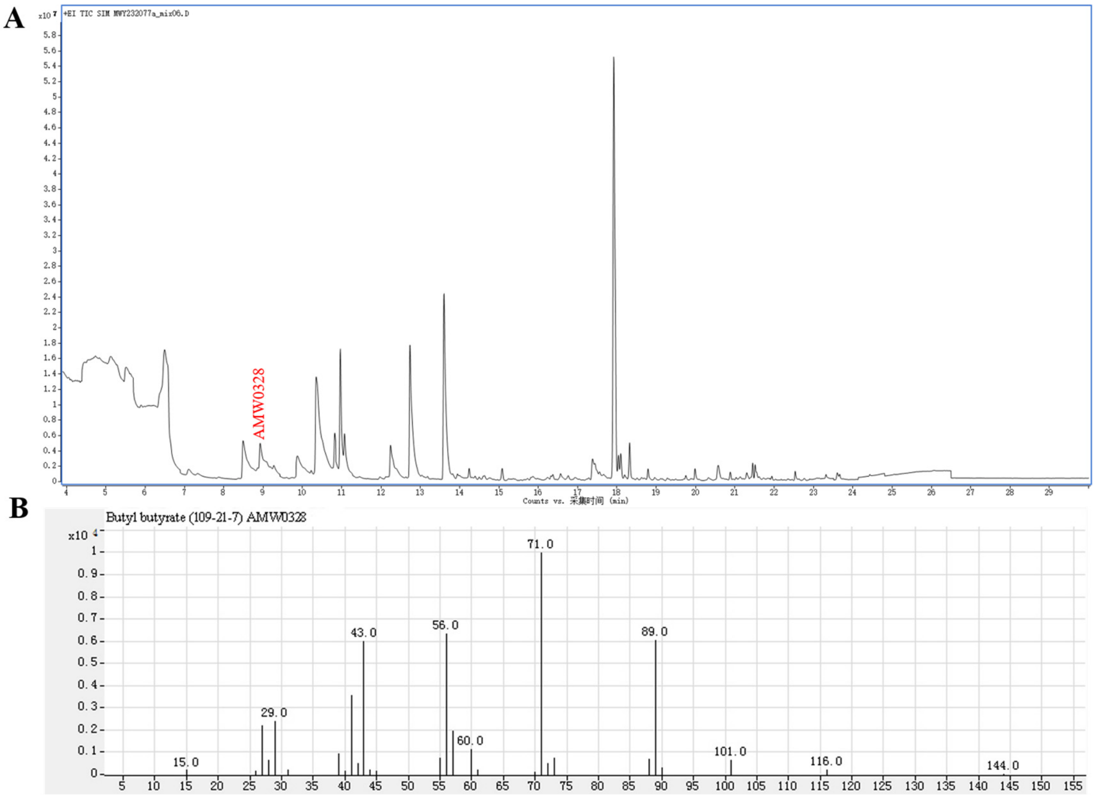

(A) The total ion current (TIC) profile from the pooled quality control (QC) samples (solvent front removed for clarity) (B) Representative mass spectrum for identification of butyl butyrate.

Supplementary Figure S3.

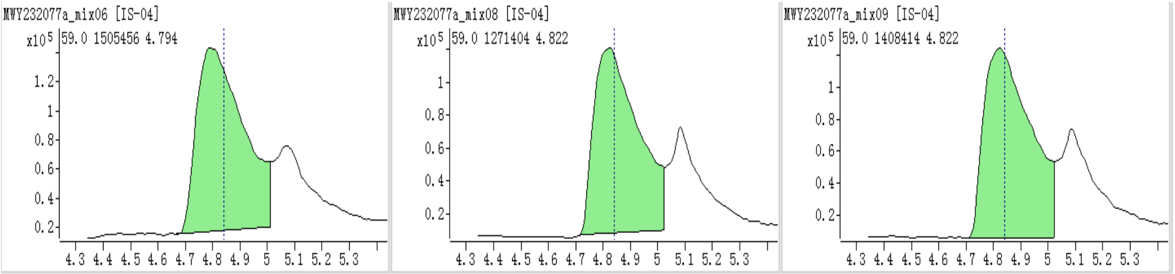

Extracted ion chromatogram (XIC) of the internal standard with integrated peak area from the QC samples.
